# A theoretical stability of mixed convection 3D Sutterby nanofluid flow due to bidirectional stretching surface

**DOI:** 10.1038/s41598-023-49798-4

**Published:** 2023-12-16

**Authors:** Humaira Yasmin, Zeeshan Zeeshan, Azzh Saad Alshehry, Abdul Hamid Ganie, Rasool Shah

**Affiliations:** 1 Department of Basic Sciences, General Administration of Preparatory Year, King Faisal University, 31982 Al-Ahsa, Saudi Arabia; 2 Department of Mathematics and Statistics, Bacha Khan University, Charsadda, KP Pakistan; 3 Department of Mathematical Sciences, Faculty of Sciences, Princess Nourah Bint Abdulrahman University, P.O. Box 84428, 11671 Riyadh, Saudi Arabia; 4 Basic Science Department, College of Science and Theoretical Studies, Saudi Electronic University, 11673 Riyadh, Saudi Arabia; 5 Department of Mathematics, Abdul Wali Khan University, Mardan, KP Pakistan

**Keywords:** Energy science and technology, Engineering, Materials science, Mathematics and computing, Nanoscience and technology, Physics

## Abstract

Host (base) fluids are unable to deliver efficient heating and cooling processes in industrial applications due to their limited heat transfer rates. Nanofluids, owing to their distinctive and adaptable thermo-physical characteristics, find a widespread range of practical applications in various disciplines of nanotechnology and heat transfer equipment. The novel effect of this study is to determine the effects of mixed convection, and activation energy on 3D Sutterby nanofluid across a bi-directional extended surface under the impact of thermophoresis diffusion and convective heat dissipation. The flow equations are simplified in terms of partial differential equations (PDEs) and altered to non-dimensional ODEs by implementing classical scaling invariants. Numerical results have been obtained via the bvp4c approach. The physical insights of crucial and relevant parameters on flow and energy profiles are analysed through plotted visuals. Some factors have multiple solutions due to shrinking sheets. So stability analysis has been adapted to analyses stable solutions. Graphical representations demonstrate the reliability and accuracy of the numerical algorithm across a variety of pertinent parameters and conditions. A comparison between existing results and previously published data shows a high degree of compatibility between the two datasets. The present study extensively explored a multitude of practical applications across a diverse spectrum of fields, including but not limited to gas turbine technology, power generation, glass manufacturing, polymer production, wire coating, chemical production, heat exchangers, geothermal engineering, and food processing.

## Introduction

In contrast to Newtonian fluids, which exhibit consistent viscosity regardless of shear stress, non-Newtonian fluids (NNFs) are distinguished by their viscosity varying due to shear stress. A prominent example of a NNF is the Maxwell fluid, extensively utilized for modeling the rheological characteristics of complex fluids like polymer, delays, and suspensions^1^. Rendering to this theoretical framework, a NNF comprises elastic as well as viscous module. The academic has exhibited significant concentration in comprehending the behaviour of NNFs, as they find applications through a broad variety of domains, comprising genetic solutions and engineering processes. The term NF flow pertains to the motion of a fluid containing deferred nano-molecules, which occurs as a response to an external pressure gradient. The inclusion of nanoparticles can lead to substantial modifications in the fluid's characteristics, such as viscosity and thermal conductivity, ultimately giving rise to complex and frequently non-conventional fluid behavior^2^. Buongiorno’s model^3^ has served as a foundational framework for the examination of heat transmission improvements in NFs. Within this context, it has been established that thermophoresis and random diffusion play pivotal roles, among other influencing factors. Prasad et al.^4^ have exemplified the behavior of radiative nanomaterial flow underneath the inspiration of the Lorentz force. Tian et al.^5^ have explored the thermal analysis of convectively heated MHD nanoliquid flow. Hayat et al.^6^ have shed light on the thermal transmission effects in convective HNFF, with a specific focus on the impact of radiation. Ahmad et al.^7^ have delved into the study of heat transmission in Maxwell nanomaterial flow exposed to a rotating porous medium. Meanwhile, Khan et al.^8^ have contributed insights into the entropy aspects of Carreau nanomaterial flow. Chu et al.^9^ have described results on the thermal properties of nanofluids incorporating viscoelastic materials, providing valuable insights into the field of thermal determination in such materials. Adnan et al.^10^ have documented their investigation into the Riga surface flow as a means of evaluating the thermal freeze valuation of NFs. Ibrahim et al.^11^ have established links related to neural networks in the context of NF-related model. Akbar and Khan^12^ have presented their work on the flow of ciliated nanofluids, taking into account the effects of viscoelastic heating. Khan et al.^13^ have provided insights into the multiple diffusion impact of NFs in the context of flat plate channel. In a related study, Khan et al.^14^ have utilized finite element method (FEM) simulations to deduce information regarding the interaction of nanoparticles in a Y-shaped obstacle. Sharma et al.^15^ have directed their research towards assessing entropy generation within Couette nanofluid flow. Additionally, Sharma et al.^16^ have observed the impact of heat and entropy generations on channel flows.

Non-Newtonian fluids are frequently favored over viscous fluids in a variety of technological and industrial contexts. These fluids, owing to their diverse characteristics, cannot be adequately characterized by a single model relationship between shear stress and shear rate. Consequently, scientists have developed numerous constitutive models tailored for non-Newtonian fluids. Sutterby^17^ introduced the constitutive equations for the non-Newtonian Sutterby fluid, which are applied in various fields such as polymer engineering, electronic cooling systems, and compact heat exchangers. Several studies pertaining to the Sutterby fluid have been documented in the scientific literature. Hayat et al.^18^ conducted a study on the peristaltic flow of the Sutterby fluid in a curved channel with consideration for radiation effects. Their research revealed that, for larger curvature parameters, the velocity distribution becomes symmetrical about the central line. Nawaz^19^ explored the behavior of hybrid nanoparticles within a Sutterby liquid and applied the Galerkin finite element method to obtain numerical solutions. Song et al.^20^ conducted an extensive analysis of bioconvection flow in a Sutterby nanoliquid under melting conditions. Their investigation revealed a significant enhancement in velocity distribution at higher Marangoni numbers. Mughanam and Almaneea^21^ undertook a numerical investigation of Sutterby nanofluid, and their results supported the use of tri-nanofluid for optimising heat transfer processes. Azam et al.^22^ reported on the bio-convection effects of Sutterby nanofluid, taking into consideration activation energy and microorganisms. They observed an increase in the microorganism population with a rise in the Peclet number. Azam^23^ introduced a model for Sutterby nanofluid that incorporates aspects of bioconvection and chemical reactions in an axisymmetric flow framework. The research indicated amplification in the microorganism population associated with higher Peclet numbers. Nadeem et al.^24^ presented a numerical assessment of Sutterby nanoliquid flow through a pipe, demonstrating an increase in fluid hotness in the presence of greater Eckert numbers. Asfour and Ibrahim^25^ employed magneto Sutterby nano liquid, incorporating a modified version of Darcy's law, and utilized a combination of dual-step difference transparent and finite approach to obtain solutions. Nadeem et al.^26^ conducted a theoretical investigation of Sutterby nano liquid, accounting for the influence of thermal slip, and obtained numerical solutions using the bvp4c function. Recent research by Du et al.^27^, Fan et al.^28^, and Qu et al.^29^ has explored diverse uses of liquids in the incidence of various nanoparticles. In recent times, numerous academics have dedicated their efforts to addressing heat transfer issues and surface density considerations in the context of both metallic and non-metallic materials. These investigations have encompassed topics such as hydrate-bearing sediments within a granulated thermodynamic structure^30^, the characterization of heat as well as pressure-dependent pore microstructures^31^, strain and distortion examination coupled with temperature fields^32^, the transportation of colloids within 2D absorptive media^33^, the study of pressure pulsations in centrifugal pumps^34^, and the analysis of energy moderation in warm electrons over their interaction with optical properties^35^. Furthermore, a substantial body of valuable work concerning fluid flow modeling is referenced in^36–40^. Akbar et al.^41^ investigated the 3D magnetized flow of viscous fluid with thermal radiation under the influence of viscous dissipations. Li et al.^42^ discussed the buoyancy effect on 3D MHD hybrid nanofluid with slip effect. Nazir et al.^43^ reported the thermal characteristics of Carreau fluid with hall forces using FE-method. Similarly, Imran et al.^44^ reported the Ellis fluid over flexible framework with entropu generation. Liu et al.^45^ gave numerical results for hybrid nanofluid over vertical channel with sorect influence.

The novel effect of this study is to explore the influences of mixed convection, and activation energy on 3D Sutterby nanofluid across a bi-directional extended surface under the impact of thermophoresis diffusion and convective heat dissipation. The flow equations are simplified in terms of PDEs and improved to non-dimensional ODEs by implementing classical scaling invariants. Numerical results have been obtained via the bvp4c approach. The physical insights of crucial and relevant parameters on flow and energy profiles are analyzed through plotted visuals. Some factors have multiple solutions due to shrinking sheets. So stability analysis has been adapted to analyses stable solutions. Graphical representations demonstrate the reliability and accuracy of the numerical algorithm across a variety of pertinent parameters and conditions. A comparison between existing results and previously published data shows a high degree of compatibility between the two datasets. The present study extensively explored a multitude of practical applications across a diverse spectrum of fields, including but not limited to gas turbine technology, power generation, glass manufacturing, polymer production, wire coating, biochemical engineering, heat exchangers, geothermal production, and food processing.

## Model description

**Figure 1:**
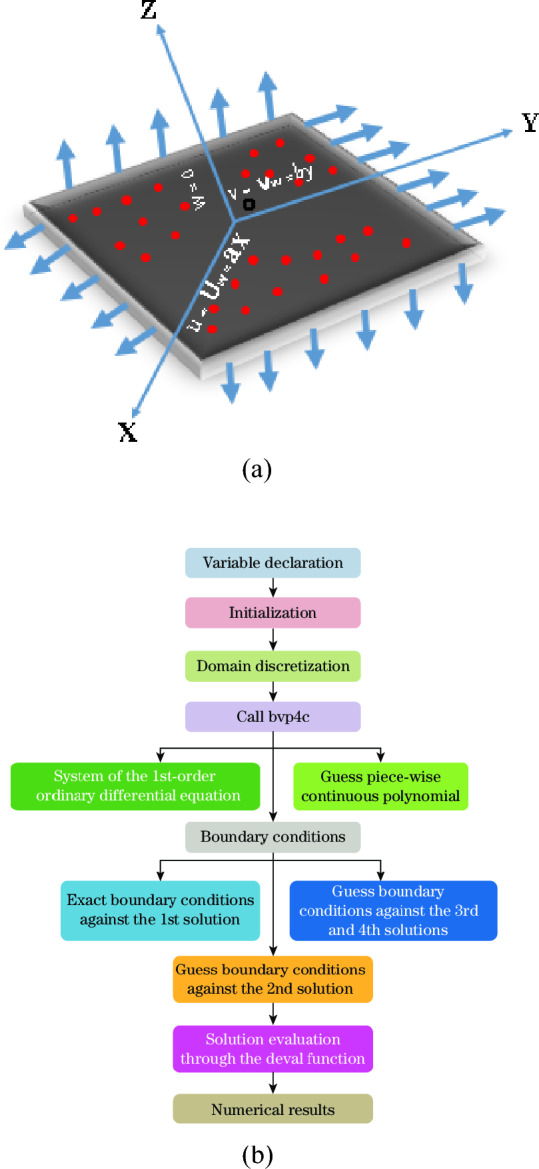
(**a**) Physical model of the problem. (**b**) Flowchart depicting the examined numerical method.

The present flow problem comprises the steady-state incompressible flow of Sutterby nanomaterials with a fixed density across a bi-directional stretchable surface along the xy-direction underneath the inspiration of viscous dissipation and variable thermal conductivity. The model incorporates the mass conservation relation, momentum and energy conservation expressions, and concentration equations. The mathematical framework considers the Arrhenius activation energy effect, mixed convection implications, and the effect of chemical processes occurring during fluid flow. The Buongiorno nanofluid model is designed to incorporate both thermophoresis and Brownian motion. Moreover, it is presumed that the host fluid is laminar and that the tiny materials are in a thermal stability state with the host fluid. The extended sheet is positioned at $z^{*} = 0$, and the fluid flow is induced by the bi-directional surface movement. The sheet is stretched along $x$- and $y$-axis with velocity $U_{w} = \lambda_{1} ax^{*}$ and $V_{w} = by^{*}$, respectively, where $a \, and \, b$ are constant with inverse time dimensions. Also it is assumed that $T_{w}^{*} ({\text{surface temperature}})$ > $T_{\infty }^{*} ({\text{ambient temperature}})$.Similarly,$C_{w}^{*} ({\text{concentration}})$ > $C_{\infty }^{*} ({\text{ambient concentration}})$. Figure 1a is provided to facilitate the clear understanding of this communication.

For Sutterby nanofluid model^17^ the stress tensor ${\varvec{\tau}}$ is achieved and formulated through the following expression:

1 $${\varvec{\tau}} = \mu \left( {\overline{\gamma } } \right){\varvec{A}}_{1} - p{\varvec{I}}$$

With viscosity^19^

2 $$\mu = \mu_{0} \left( {\frac{{\sinh^{ - 1} \left( {\beta \overline{\gamma } } \right)}}{{\beta \overline{\gamma } }}} \right)^{n}$$

here $\beta$ represents the material's time constant, $n$ stands for power law index, and $\mu_{0}$ denotes the viscosity at zero shear rate. Substituting Eq. (2) into Eq. (1), we obtain:

3 $${\varvec{\tau}} = \mu_{0} \left( {\frac{{\sinh^{ - 1} \left( {\beta \overline{\gamma } } \right)}}{{\beta \overline{\gamma } }}} \right)^{n} {\varvec{A}}_{1} - p{\varvec{I}}$$

For Sutterby fluid model the shear rate $\overline{\gamma }$ is formulated as

4 $$\overline{\gamma } = \sqrt {\left( {0.5} \right)tr\left( {{\varvec{A}}_{1}^{2} } \right)}$$

In the context of 3D, incompressible, and steady nanofluid flow, the basic flow equations are^19^:

5 $$\user2{V = }\left( {u^{*} , \, v^{*} , \, w^{*} } \right), \, T^{*} = T^{*} \left( {x^{*} ,y^{*} ,z^{*} } \right), \, C^{*} = C^{*} \left( {x^{*} ,y^{*} ,z^{*} } \right)$$

Here are the basis expressions that describe the Sutterby nanofluid flow problem, comprising nonlinear resulting expressions categorized into mass conservation, momentum, energy, and concentration expressions are stated as^17,19^:

6 $$\frac{{\partial u^{*} }}{{\partial x^{*} }} + \frac{{\partial v^{*} }}{{\partial y^{*} }} + \frac{{\partial w^{*} }}{{\partial z^{*} }} = 0$$

7 $$\begin{aligned} & u^{*} \frac{{\partial u^{*} }}{{\partial x^{*} }} + v^{*} \frac{{\partial u^{*} }}{{\partial y^{*} }} + w^{*} \frac{{\partial u^{*} }}{{\partial z^{*} }} = \nu \left( {1 - \frac{1}{6}\beta^{2} \frac{{\partial u^{*} }}{{\partial z^{*} }}} \right)^{n} \left( {\frac{{\partial^{2} u^{*} }}{{\partial z^{*2} }}} \right) \\ & \quad - \frac{1}{6}\left( {\beta^{2} \nu n} \right)\left( {1 - \frac{1}{6}\beta^{2} \frac{{\partial u^{*} }}{{\partial z^{*} }}} \right)^{n - 1} \left( {\frac{{\partial u^{*} }}{{\partial z^{*} }}} \right)\left( {\frac{{\partial^{2} u^{*} }}{{\partial z^{*2} }}} \right) + g^{*} \beta_{{T^{*} }} \left( {T^{*} - T_{\infty } } \right) + g^{*} \beta_{{C^{*} }} \left( {C^{*} - C_{\infty } } \right) \\ \end{aligned}$$

8 $$\begin{aligned} & u^{*} \frac{{\partial v^{*} }}{{\partial x^{*} }} + v^{*} \frac{{\partial v^{*} }}{{\partial y^{*} }} + w^{*} \frac{{\partial v^{*} }}{{\partial z^{*} }} = \nu \left( {1 - \frac{1}{6}\beta^{2} \frac{{\partial v^{*} }}{{\partial z^{*} }}} \right)^{n} \left( {\frac{{\partial^{2} v^{*} }}{{\partial z^{*2} }}} \right) \\ & \quad - \frac{1}{6}\left( {\beta^{2} \nu n} \right)\left( {1 - \frac{1}{6}\beta^{2} \frac{{\partial v^{*} }}{{\partial z^{*} }}} \right)^{n - 1} \left( {\frac{{\partial u^{*} }}{{\partial z^{*} }}} \right)\left( {\frac{{\partial^{2} v^{*} }}{{\partial z^{*2} }}} \right) \\ \end{aligned}$$

9 $$\begin{aligned} & u^{*} \frac{{\partial T^{*} }}{{\partial x^{*} }} + v^{*} \frac{{\partial T^{*} }}{{\partial y^{*} }} + w^{*} \frac{{\partial T^{*} }}{{\partial z^{*} }} = \alpha^{*} \frac{{\partial^{2} T^{*} }}{{\partial z^{*2} }} + \frac{1}{{C_{p} }}\left( {1 - \frac{1}{6}\beta^{2} \frac{{\partial u^{*} }}{{\partial z^{*} }}} \right)^{n} \left( {\frac{{\partial u^{*} }}{{\partial z^{*} }}} \right)^{2} \\ & \quad + \tau^{*} \left( {D_{B} \left( {\frac{{\partial C^{*} }}{{\partial z^{*} }}\frac{{\partial T^{*} }}{{\partial z^{*} }}} \right) + \frac{{D_{T} }}{{T_{\infty }^{*} }}\left( {\frac{{\partial T^{*} }}{{\partial z^{*} }}\frac{{\partial T^{*} }}{{\partial z^{*} }}} \right)} \right) \\ \end{aligned}$$

10 $$u^{*} \frac{{\partial C^{*} }}{{\partial x^{*} }} + v^{*} \frac{{\partial C^{*} }}{{\partial y^{*} }} + w^{*} \frac{{\partial C^{*} }}{{\partial z^{*} }} = D_{B} \left( {\frac{{\partial^{2} C^{*} }}{{\partial z^{*2} }}} \right) + \frac{{D_{T} }}{{T_{\infty }^{*} }}\left( {\frac{{\partial^{2} T^{*} }}{{\partial z^{*2} }}} \right) - \left( {Kr} \right)^{2} \left( {C^{*} - C_{\infty }^{*} } \right)\left( {\frac{{T^{*} }}{{T_{\infty }^{*} }}} \right)^{m} \exp \left( {\frac{Ea}{{K_{1} T^{*} }}} \right)$$

where $a^{*}$ shows thermal diffusivity, $D_{{T^{*} }}$ thermophoretic diffusion coefficient, $K_{1}$ Boltzmann constant, $\beta_{{T^{*} }}$ thermal expansion coefficient, $C_{p}$ specific heat, $\nu$ viscosity, $Ea$ Arrhenius energy factor, $g$ gravitational force and $D_{B}$ Brownian effect. Consequently, the boundary conditions pertaining to the geometry of the problem are presented underneath^42^:

11 $$\begin{aligned} & u^{*} = U_{w} = \lambda_{1} ax^{*} , \, v^{*} = by^{*} , \, w^{*} = 0, \, T^{*} = T_{w}^{*} , \, C^{*} = C_{w}^{*} \;at\;z^{*} = 0 \\ & u^{*} = 0,\;v^{*} = 0,\;T^{*} = T_{\infty }^{*} ,\;C^{*} = C_{\infty }^{*} \;as\;z^{*} = \infty \\ \end{aligned}$$

In mathematics, it is an extensive exercise to streamline a set of PDEs through the application of similarity transformations. This method entails the introduction of new variables connected to the initial variables by means of a scaling factor. Consequently, the subsequent transformations are frequently employed^19^:

12 $$\begin{aligned} & \eta = z^{*} \left( {\frac{a}{\upsilon }} \right)^{0.5} ,\;u^{*} = ax^{*} u_{0}^{\prime } \left( \eta \right),\;v^{*} = ay^{*} u_{1}^{\prime } \left( \eta \right),\;w^{*} = - 2\left( {a\upsilon } \right)^{0.5} \left[ {u_{0} \left( \eta \right) + u_{1} \left( \eta \right)} \right], \\ & g_{0} \left( \eta \right) = \frac{{T^{*} - T_{\infty }^{*} }}{{T_{w}^{*} - T_{\infty }^{*} }}, \, h_{0} \left( \eta \right) = \frac{{C^{*} - C_{\infty }^{*} }}{{C_{w}^{*} - C_{\infty }^{*} }}. \\ \end{aligned}$$

With the assistance of Eq. (12) and ensuring compliance with the continuity Eq. (6), the resulting highly nonlinear expressions can be formulated as follows:

13 $$\left( {1 - \frac{1}{6}\beta_{1} u_{0}^{\prime \prime } } \right)^{n} u_{0}^{\prime \prime \prime } - \frac{1}{6}n\beta_{1} \left( {1 - \frac{1}{6}\beta_{1} u_{0}^{\prime \prime } } \right)^{n - 1} u_{0}^{\prime \prime } u_{0}^{\prime \prime \prime } + \left( {u_{0} + u_{1} } \right)u_{0}^{\prime \prime } - \left( {u_{0}^{\prime } } \right)^{2} + \lambda \left( {g_{0} + \alpha h_{0} } \right) = 0$$

14 $$\left( {1 - \frac{1}{6}\beta_{2} u_{1}^{\prime \prime } } \right)^{n} u_{1}^{\prime \prime \prime } - \frac{1}{6}n\beta_{2} \left( {1 - \frac{1}{6}\beta_{2} u_{1}^{\prime \prime } } \right)^{n - 1} u_{1}^{\prime \prime } u_{1}^{\prime \prime \prime } + \left( {u_{0} + u_{1} } \right)u_{1}^{\prime \prime } - \left( {u_{1}^{\prime } } \right)^{2} = 0$$

15 $$\frac{1}{\Pr }g_{0}^{\prime \prime } + \left( {u_{0} + u_{1} } \right)g_{0}^{\prime } + Ec\left( {1 - \frac{1}{6}\beta_{1} u_{0}^{\prime \prime } } \right)^{n} \left( {u_{0}^{\prime \prime } } \right)^{2} + Nb\left( {g_{0}^{\prime } h_{0}^{\prime } + \frac{Nt}{{Nb}}\left( {g_{0}^{\prime } } \right)^{2} } \right) = 0$$

16 $$\frac{1}{Sc}h_{0}^{\prime \prime } + \left( {u_{0} + u_{1} } \right)h_{0}^{\prime } - \sigma \left( {1 + \delta g_{0} } \right)^{m} h_{0} \exp \left( {\frac{ - E}{{1 + \delta g_{0} }}} \right) + \left( \frac{1}{Sc} \right)\left( {\frac{Nt}{{Nb}}} \right)g_{0}^{\prime \prime } = 0.$$

Moreover, the appropriate boundary conditions are expressed as follows:

17 $$\begin{aligned} & u_{0} = 0,\;u_{0}^{\prime } = \lambda_{1} ,\;u_{1} = 0,\;u_{1}^{\prime } = \gamma ,\;g_{0} = 1,\;h_{0} = 1\;at\;\eta = 0 \\ & u_{0}^{\prime } = 0,\;u_{1}^{\prime } = 0,\;g_{0} = 0,\;h_{0} = 0\;as\;\eta = \infty . \\ \end{aligned}$$

## Non-dimensional parameters

It is looked over that the nonlinear system stated earlier is predominantly measured and controlled by the following non-dimensional data involved in Eqs. (13)–(17) are simulated and enumerated beneath:

$\left( {\lambda = \frac{{\beta_{{T^{*} }} g\left( {T_{w}^{*} - T_{\infty }^{*} } \right)}}{{a^{2} x^{*} }}} \right)$ mixed convection parameter, $\left( {\beta_{1} = \beta^{2} ax^{*} \sqrt {\frac{a}{\nu }} } \right)$ Sutterby fluid parameter, $\left( {E = \frac{Ea}{{T_{\infty }^{*} K_{1} }}} \right)$ activation energy parameter, $\left( {\beta_{2} = \beta^{2} ay^{*} \sqrt {\frac{a}{\nu }} } \right)$ Sutterby fluid parameter, $\left( {Nb = \frac{{\tau^{*} D_{B} \left( {C_{w}^{*} - C_{\infty }^{*} } \right)}}{\nu }} \right)$ Brownian behavior particles $\left( {Ec = \frac{{\left( {ax^{*} } \right)^{2} }}{{C_{p} \left( {T_{w}^{*} - T_{\infty }^{*} } \right)}}} \right)$ Eckert number, $\left( {Nt = \frac{{\tau^{*} D_{{T^{*} }} \left( {T_{w}^{*} - T_{\infty }^{*} } \right)}}{{\nu T_{\infty }^{*} }}} \right)$ thermophoresis parameter, $\left( {\Pr = \frac{\upsilon }{{a^{*} }}} \right)$ Prandtl number, $\left( {\alpha = \frac{{\beta_{{C^{*} }} \left( {C_{w}^{*} - C_{\infty }^{*} } \right)}}{{\beta_{{T^{*} }} \left( {T_{w}^{*} - T_{\infty }^{*} } \right)}}} \right)$ buoyancy parameter, $\left( {\gamma = \frac{a}{b}} \right)$ ratio of stretching rates, $\left( {\delta = \frac{{T_{w}^{*} - T_{\infty }^{*} }}{{T_{\infty }^{*} }}} \right)$ temperature difference parameter, $\left( {Sc = \frac{\nu }{{D_{B} }}} \right)$ Schmidt number.

## Quantities of engineering concern

In the context of the present analysis, the essential physical quantities of interest, which include the surface friction factor in both the directions, as well as wall-related heat and mass flow rates.

The skin force coefficient in the x*-*direction* is represented as $Cf_{{x^{*} }}$ and can be defined as follows:

18 $$Cf_{{x^{*} }} = \left. {\frac{1}{\rho }\frac{{\tau_{{x^{*} z^{*} }} }}{{\left( {ax^{*} } \right)^{2} }}} \right|_{{z^{*} = 0}}$$

The determination of $\tau_{{x^{*} z^{*} }}$, which denotes the respective wall stress in the x*-*direction* for nanofluid, is established through the following relation:

19 $$\tau_{{x^{*} z^{*} }} = \left. {\mu_{0} \left( {1 - \frac{1}{6}\beta^{2} \frac{{\partial u^{*} }}{{\partial z^{*} }}} \right)^{n} \frac{{\partial u^{*} }}{{\partial z^{*} }}} \right|_{{z^{*} = 0}}$$

The following dimensionless expression is obtained by inserting Eq. (19) into Eq. (18):

20 $$Cf_{x} = \frac{1}{{\sqrt {{\text{Re}}_{x} } }}\left( {1 - \frac{1}{6}\beta_{1} u_{0}^{\prime \prime } \left( 0 \right)} \right)^{n} u_{0}^{\prime \prime } \left( 0 \right)$$

here, ${\text{Re}}_{x} = {{x^{*} U_{w} } \mathord{\left/ {\vphantom {{x^{*} U_{w} } \nu }} \right. \kern-0pt} \nu }$ represents the Reynolds number in the x*-*direction*.

The correlation for the drag force in the y*-*direction*, denoted as $Cf_{{y^{*} }}$, is as follows:

21 $$Cf_{{y^{*} }} = \left. {\frac{1}{\rho }\frac{{\tau_{{w^{*} y^{*} }} }}{{V_{w}^{2} }}} \right|_{{z^{*} = 0}}$$

the shear stress in the y*-*direction*, denoted as $\tau_{{y^{*} z^{*} }}$, is expressed as follows:

22 $$\tau_{{y^{*} z^{*} }} = \left. {\mu_{0} \left( {1 - \frac{1}{6}\beta^{2} \frac{{\partial v^{*} }}{{\partial z^{*} }}} \right)^{n} \frac{{\partial v^{*} }}{{\partial z^{*} }}} \right|_{{z^{*} = 0}}$$

By incorporating Eq. (22) into Eq. (21), it uncovers the ensuing dimensionless form in the normal direction:

23 $$Cf_{y} = \frac{1}{{\sqrt \gamma \sqrt {{\text{Re}}_{y} } }}\left( {1 - \frac{1}{6}\beta_{2} u_{1}^{\prime \prime } \left( 0 \right)} \right)^{n} u_{1}^{\prime \prime } \left( 0 \right)$$

here ${\text{Re}}_{y} = {{y^{*} V_{w} } \mathord{\left/ {\vphantom {{y^{*} V_{w} } \nu }} \right. \kern-0pt} \nu }$ represents the Reynolds number in the y*-*direction*.

The expression for $Nu_{{x^{*} }}$, which symbolizes wall heat transfer, can be formulated in the following manner:

24 $$Nu_{{x^{*} }} = \left. {\frac{{x^{*} q_{w} }}{{k\left( {T_{w}^{*} - T_{\infty }^{*} } \right)}}} \right|_{{z^{*} = 0}}$$

The heat transfers at the wall, represented as $q_{{w^{*} }}$, computed as follows:

25 $$q_{{w^{*} }} = \left. { - k\frac{{\partial T^{*} }}{{\partial z^{*} }}} \right|_{{z^{*} = 0}}$$

By substituting Eqs. (25) to (24), we obtain the following result:

26 $$Nu_{x} = - \frac{1}{{\sqrt {{\text{Re}}_{x} } }}g_{0}^{\prime } \left( 0 \right)$$

The representation of wall mass transfer, indicated by $Sh_{{x^{*} }}$, can be described as follows:

27 $$Sh_{{x^{*} }} = \left. {\frac{{x^{*} q_{m} }}{{D_{B} \left( {C_{w}^{*} - C_{\infty }^{*} } \right)}}} \right|_{{z^{*} = 0}}$$

The wall mass flux, denoted as $q_{m}$, is defined as follows:

28 $$q_{m} = \left. { - D_{B} \frac{{\partial C^{*} }}{{\partial z^{*} }}} \right|_{{z^{*} = 0}}$$

Substituting Eq. (28) within Eq. (29) yields.

29 $$Sh_{{x^{*} }} = - \frac{1}{{\sqrt {{\text{Re}}_{x} } }}h_{0}^{\prime } \left( 0 \right)$$

## Stability analysis

The resulting nonlinear differential equations are elucidated numerically via the bvp4c scheme along with the shooting technique. Some parameters exhibit duality, so stability scrutiny is implemented for the present study. Dual solutions occur due to suction and injection. For this purpose, it is significant to find a stability analysis of the problem that is both stable and physically feasible. The procedure adopted for stability purposes is the same as discussed by Merkin^46^ and Weidman et al.^47^. The time-dependent flow characteristics (1)–(6) are given below

30 $$\begin{aligned} & \frac{{\partial u^{*} }}{{\partial t^{*} }} + u^{*} \frac{{\partial u^{*} }}{{\partial x^{*} }} + v^{*} \frac{{\partial u^{*} }}{{\partial y^{*} }} + w^{*} \frac{{\partial u^{*} }}{{\partial z^{*} }} = \nu \left( {1 - \frac{1}{6}\beta^{2} \frac{{\partial u^{*} }}{{\partial z^{*} }}} \right)^{n} \left( {\frac{{\partial^{2} u^{*} }}{{\partial z^{*2} }}} \right) \\ & \quad - \;\frac{1}{6}\left( {\beta^{2} \nu n} \right)\left( {1 - \frac{1}{6}\beta^{2} \frac{{\partial u^{*} }}{{\partial z^{*} }}} \right)^{n - 1} \left( {\frac{{\partial u^{*} }}{{\partial z^{*} }}} \right)\left( {\frac{{\partial^{2} u^{*} }}{{\partial z^{*2} }}} \right) + g^{*} \beta_{{T^{*} }} \left( {T^{*} - T_{\infty } } \right) + g^{*} \beta_{{C^{*} }} \left( {C^{*} - C_{\infty } } \right) \\ \end{aligned}$$

31 $$\begin{aligned} & \frac{{\partial v^{*} }}{{\partial t^{*} }} + u^{*} \frac{{\partial v^{*} }}{{\partial x^{*} }} + v^{*} \frac{{\partial v^{*} }}{{\partial y^{*} }} + w^{*} \frac{{\partial v^{*} }}{{\partial z^{*} }} = \nu \left( {1 - \frac{1}{6}\beta^{2} \frac{{\partial v^{*} }}{{\partial z^{*} }}} \right)^{n} \left( {\frac{{\partial^{2} v^{*} }}{{\partial z^{*2} }}} \right) \\ & \quad - \;\frac{1}{6}\left( {\beta^{2} \nu n} \right)\left( {1 - \frac{1}{6}\beta^{2} \frac{{\partial v^{*} }}{{\partial z^{*} }}} \right)^{n - 1} \left( {\frac{{\partial u^{*} }}{{\partial z^{*} }}} \right)\left( {\frac{{\partial^{2} v^{*} }}{{\partial z^{*2} }}} \right) \\ \end{aligned}$$

32 $$\begin{aligned} & \frac{{\partial T^{*} }}{{\partial t^{*} }} + u^{*} \frac{{\partial T^{*} }}{{\partial x^{*} }} + v^{*} \frac{{\partial T^{*} }}{{\partial y^{*} }} + w^{*} \frac{{\partial T^{*} }}{{\partial z^{*} }} = \alpha^{*} \frac{{\partial^{2} T^{*} }}{{\partial z^{*2} }} + \frac{1}{{C_{p} }}\left( {1 - \frac{1}{6}\beta^{2} \frac{{\partial u^{*} }}{{\partial z^{*} }}} \right)^{n} \left( {\frac{{\partial u^{*} }}{{\partial z^{*} }}} \right)^{2} \\ & \quad + \;\tau^{*} \left( {D_{B} \left( {\frac{{\partial C^{*} }}{{\partial z^{*} }}\frac{{\partial T^{*} }}{{\partial z^{*} }}} \right) + \frac{{D_{T} }}{{T_{\infty }^{*} }}\left( {\frac{{\partial T^{*} }}{{\partial z^{*} }}\frac{{\partial T^{*} }}{{\partial z^{*} }}} \right)} \right) \\ \end{aligned}$$

33 $$\frac{{\partial C^{*} }}{{\partial t^{*} }} + u^{*} \frac{{\partial C^{*} }}{{\partial x^{*} }} + v^{*} \frac{{\partial C^{*} }}{{\partial y^{*} }} + w^{*} \frac{{\partial C^{*} }}{{\partial z^{*} }} = D_{B} \left( {\frac{{\partial^{2} C^{*} }}{{\partial z^{*2} }}} \right) + \frac{{D_{T} }}{{T_{\infty }^{*} }}\left( {\frac{{\partial^{2} T^{*} }}{{\partial z^{*2} }}} \right) - \left( {Kr} \right)^{2} \left( {C^{*} - C_{\infty }^{*} } \right)\left( {\frac{{T^{*} }}{{T_{\infty }^{*} }}} \right)^{m} \exp \left( {\frac{Ea}{{K_{1} T^{*} }}} \right).$$

To find the stability of the solutions, the following transformations are introduced to transform the system to unsteady ODEs as explained by Marken^46^ and Wiedman et al.^47^.

34 $$\begin{aligned} & \eta = z^{*} \left( {\frac{a}{\upsilon }} \right)^{0.5} , \, u^{*} (x^{*} ,t^{*} ) = ax^{*} u_{0}{\prime} \left( {\eta ,t} \right), \, v^{*} (x^{*} ,t^{*} ) = ay^{*} u_{1}{\prime} \left( {\eta ,t} \right), \\ & w^{*} (x^{*} ,t^{*} ) = - 2\left( {a\upsilon } \right)^{0.5} \left[ {u_{0} \left( {\eta ,t} \right) + u_{1} \left( {\eta ,t} \right)} \right],g_{0} \left( {\eta ,t} \right) = \frac{{T^{*} - T_{\infty }^{*} }}{{T_{w}^{*} - T_{\infty }^{*} }}, \\ & h_{0} \left( {\eta ,t} \right) = \frac{{C^{*} - C_{\infty }^{*} }}{{C_{w}^{*} - C_{\infty }^{*} }},\;\tau = ct^{*} . \\ \end{aligned}$$

Equation (20) in Eqs. (16)–(19), we get

35 $$\begin{aligned} & \left( {1 - \frac{1}{6}\beta_{1} \frac{{\partial^{2} u_{0} }}{{\partial \eta^{2} }}} \right)^{n} \frac{{\partial^{3} u_{0} }}{{\partial \eta^{3} }} - \frac{1}{6}n\beta_{1} \left( {1 - \frac{1}{6}\beta_{1} \frac{{\partial^{2} u_{0} }}{{\partial \eta^{2} }}} \right)^{n - 1} \frac{{\partial^{2} u_{0} }}{{\partial \eta^{2} }}\frac{{\partial^{3} u_{0} }}{{\partial \eta^{3} }} + \left( {u_{0} + u_{1} } \right)\frac{{\partial^{2} u_{0} }}{{\partial \eta^{2} }} - \left( {\frac{{\partial u_{0} }}{\partial \eta }} \right)^{2} \\ & \quad + \;\lambda \left( {g_{0} + \alpha h_{0} } \right) - \frac{{\partial^{2} u_{0} }}{\partial \eta \partial \tau } = 0 \\ \end{aligned}$$

36 $$\begin{aligned} & \left( {1 - \frac{1}{6}\beta_{2} \frac{{\partial^{2} u_{1} }}{{\partial \eta^{2} }}} \right)^{n} \frac{{\partial^{3} u_{1} }}{{\partial \eta^{3} }} - \frac{1}{6}n\beta_{2} \left( {1 - \frac{1}{6}\beta_{2} \frac{{\partial^{2} u_{1} }}{{\partial \eta^{2} }}} \right)^{n - 1} \frac{{\partial^{2} u_{1} }}{{\partial \eta^{2} }}\frac{{\partial^{3} u_{1} }}{{\partial \eta^{3} }} + \left( {u_{0} + u_{1} } \right)\frac{{\partial^{2} u_{1} }}{{\partial \eta^{2} }} - \left( {\frac{{\partial u_{1} }}{\partial \eta }} \right)^{2} \\ & \quad - \;\frac{{\partial^{2} u_{1} }}{\partial \eta \partial \tau } = 0 \\ \end{aligned}$$

37 $$\frac{1}{\Pr }\frac{{\partial^{2} g_{0} }}{{\partial \eta^{2} }} + \left( {u_{0} + u_{1} } \right)\frac{{\partial g_{0} }}{\partial \eta } + Ec\left( {1 - \frac{1}{6}\beta_{1} \frac{{\partial^{2} u_{0} }}{{\partial \eta^{2} }}} \right)^{n} \left( {\frac{{\partial^{2} u_{0} }}{{\partial \eta^{2} }}} \right)^{2} - \frac{{\partial^{2} u_{0} }}{\partial \eta \partial \tau } + Nb\left( {\frac{{\partial g_{0} }}{\partial \eta }\frac{{\partial h_{0} }}{\partial \eta } + \frac{Nt}{{Nb}}\left( {\frac{{\partial g_{0} }}{\partial \eta }} \right)^{2} } \right) = 0$$

38 $$\frac{1}{Sc}\frac{{\partial^{2} h_{0} }}{{\partial \eta^{2} }} + \left( {u_{0} + u_{1} } \right)\frac{{\partial h_{0} }}{\partial \eta } - \sigma \left( {1 + \delta g_{0} } \right)^{m} h_{0} \exp \left( {\frac{ - E}{{1 + \delta g_{0} }}} \right) + \left( \frac{1}{Sc} \right)\left( {\frac{Nt}{{Nb}}} \right)\frac{{\partial^{2} h_{0} }}{{\partial \eta^{2} }} - \frac{{\partial^{2} h_{0} }}{\partial \eta \partial \tau } = 0.$$

Moreover, the appropriate boundary conditions are expressed as follows:

39 $$\begin{aligned} & u_{0} (\eta ,\tau ) = 0, \, \frac{{\partial u_{0} }}{\partial \eta }(\eta ,\tau ) = \lambda_{1} , \, u_{1} (\eta ,\tau ) = 0, \, \frac{{\partial u_{1} }}{\partial \eta }(\eta ,\tau ) = \gamma , \, g_{0} (\eta ,\tau ) = 1, \, h_{0} (\eta ,\tau ) = 1 \, at \, \eta = 0 \\ & \frac{{\partial u_{0} }}{\partial \eta }(\eta ,\tau ) = 0 \, , \, \frac{{\partial u_{1} }}{\partial \eta }(\eta ,\tau ) = 0 \, , \, g_{0} (\eta ,\tau ) = 0, \, h_{0} (\eta ,\tau ) = 0 \, as \, \eta = \infty . \\ \end{aligned}$$

Using the following expression, the stability solutions are inquired for the steady movement $u_{0} (\eta ) = J_{0} (\eta ) \, , \, u_{1} (\eta ) = H_{0} (\eta ) \, , \, g_{0} (\eta ) = I_{0} (\eta ),{\text{ and }}h_{0} (\eta ) = F_{0} (\eta )$ that fulfills the far-field boundary conditions as reported by Merkin^46^ and Wiedman et al.^47^.

40 $$\begin{aligned} & u_{0} (\eta ,\tau ) = J_{0} (\eta ) + e^{ - \gamma \tau } J{(}\eta ,\tau {) }, \, u_{1} (\eta ) = H_{0} (\eta ) + e^{ - \gamma \tau } H{(}\eta ,\tau {)}, \, \\ & g_{0} (\eta ) = I_{0} (\eta ) + e^{ - \gamma \tau } I{(}\eta ,\tau {)},{\text{ and }}h_{0} (\eta ) = F_{0} (\eta ) + e^{ - \gamma \tau } F{(}\eta ,\tau {)}{\text{.}} \\ \end{aligned}$$

Unknown parameters, $\gamma$ is the smallest eigenvalues.

In view of (40), Eqs. (35)–(39) become

41 $$\begin{aligned} & \left( {1 - \frac{1}{6}\beta_{1} \frac{{\partial^{2} J}}{{\partial \eta^{2} }}} \right)^{n} \frac{{\partial^{3} J}}{{\partial \eta^{3} }} - \frac{1}{6}n\beta_{1} \left( {1 - \frac{1}{6}\beta_{1} \frac{{\partial^{2} J}}{{\partial \eta^{2} }}} \right)^{n - 1} \frac{{\partial^{2} J}}{{\partial \eta^{2} }}\frac{{\partial^{3} J}}{{\partial \eta^{3} }} + \left( {J_{0} + H_{0} } \right)\frac{{\partial^{2} J}}{{\partial \eta^{2} }} - \left( {\frac{\partial J}{{\partial \eta }}} \right)^{2} \\ & \quad + \;\lambda \left( {I_{0} + \alpha F_{0} } \right) - \gamma J + \frac{\partial J}{{\partial \tau }} = 0, \\ \end{aligned}$$

42 $$\begin{aligned} & \left( {1 - \frac{1}{6}\beta_{2} \frac{{\partial^{2} H}}{{\partial \eta^{2} }}} \right)^{n} \frac{{\partial^{3} H}}{{\partial \eta^{3} }} - \frac{1}{6}n\beta_{2} \left( {1 - \frac{1}{6}\beta_{2} \frac{{\partial^{2} H}}{{\partial \eta^{2} }}} \right)^{n - 1} \frac{{\partial^{2} H}}{{\partial \eta^{2} }}\frac{{\partial^{3} H}}{{\partial \eta^{3} }} + \left( {J_{0} + H_{0} } \right)\frac{{\partial^{2} H}}{{\partial \eta^{2} }} - \left( {\frac{\partial H}{{\partial \eta }}} \right)^{2} \\ & \quad - \gamma H + \frac{\partial H}{{\partial \tau }} = 0, \\ \end{aligned}$$

43 $$\frac{1}{\Pr }\frac{{\partial^{2} I}}{{\partial \eta^{2} }} + \left( {J_{0} + H_{0} } \right)\frac{\partial I}{{\partial \eta }} + Ec\left( {1 - \frac{1}{6}\beta_{1} \frac{{\partial^{2} J}}{{\partial \eta^{2} }}} \right)^{n} \left( {\frac{{\partial^{2} J}}{{\partial \eta^{2} }}} \right)^{2} - \gamma I + \frac{\partial I}{{\partial \tau }} + Nb\left( {\frac{\partial I}{{\partial \eta }}\frac{\partial F}{{\partial \eta }} + \frac{Nt}{{Nb}}\left( {\frac{\partial I}{{\partial \eta }}} \right)^{2} } \right) = 0$$

44 $$\frac{1}{Sc}\frac{{\partial^{2} F}}{{\partial \eta^{2} }} + \left( {J_{0} + } \right)H_{0} \frac{\partial F}{{\partial \eta }} - \sigma \left( {1 + \delta I_{0} } \right)^{m}_{0} F_{0} \exp \left( {\frac{ - E}{{1 + \delta F_{0} }}} \right) + \left( \frac{1}{Sc} \right)\left( {\frac{Nt}{{Nb}}} \right)\frac{{\partial^{2} F}}{{\partial \eta^{2} }} - \gamma F + \frac{\partial F}{{\partial \tau }} = 0$$

The boundary conditions are

45 $$\begin{aligned} & J_{0} (\eta ,\tau ) = 0,\;\frac{\partial J}{{\partial \eta }}(\eta ,\tau ) = \lambda_{1} ,\;H_{0} (\eta ,\tau ) = 0,\;\frac{\partial H}{{\partial \eta }}(\eta ,\tau ) = \gamma ,\;I_{0} (\eta ,\tau ) = 1,\;F_{0} (\eta ,\tau ) = 1\;at\;\eta = 0 \\ & \frac{\partial J}{{\partial \eta }}(\eta ,\tau ) = 0,\;\frac{\partial H}{{\partial \eta }}(\eta ,\tau ) = 0,\;I_{0} (\eta ,\tau ) = 0,\;F_{0} (\eta ,\tau ) = 0\;as\;\eta = \infty . \\ \end{aligned}$$

The steady state linear eigenvalues problem of Eq. (41)–(44) is given below

46 $$\begin{aligned} & \left( {1 - \frac{1}{6}\beta_{1} J^{\prime \prime } } \right)^{n} J^{\prime \prime \prime } - \frac{1}{6}n\beta_{1} \left( {1 - \frac{1}{6}\beta_{1} J^{\prime \prime } } \right)^{n - 1} J^{\prime \prime } J^{\prime \prime \prime } + \left( {J + H} \right)J^{\prime \prime } - \left( {J^{\prime } } \right)^{2} \\ & \quad + \lambda \left( {I + \alpha F} \right) - \gamma J = 0, \\ \end{aligned}$$

47 $$\begin{aligned} & \left( {1 - \frac{1}{6}\beta_{2} H^{\prime \prime } } \right)^{n} H^{\prime \prime \prime } - \frac{1}{6}n\beta_{2} \left( {1 - \frac{1}{6}\beta_{2} H^{\prime \prime } } \right)^{n - 1} H^{\prime \prime } H^{\prime \prime \prime } + \left( {J + H} \right)H^{\prime \prime } - \left( {H^{\prime } } \right)^{2} \\ & \quad - \gamma H = 0, \\ \end{aligned}$$

48 $$\frac{1}{\Pr }I^{\prime \prime } + \left( {J + H} \right)I + Ec\left( {1 - \frac{1}{6}\beta_{1} J^{\prime \prime } } \right)^{n} \left( {J^{\prime \prime } } \right)^{2} - \gamma I + Nb\left( {I^{\prime } F^{\prime } + \frac{Nt}{{Nb}}\left( {I^{\prime } } \right)^{2} } \right) = 0$$

49 $$\frac{1}{Sc}F^{\prime \prime } + \left( {J + } \right)HF^{\prime } - \sigma \left( {1 + \delta I} \right)^{m} F\exp \left( {\frac{ - E}{{1 + \delta F}}} \right) + \left( \frac{1}{Sc} \right)\left( {\frac{Nt}{{Nb}}} \right)F^{\prime \prime } - \gamma F = 0$$

with

50 $$\begin{gathered} J(\eta ,\tau ) = 0, \, J^{\prime}(\eta ,\tau ) = \lambda_{1} , \, H(\eta ,\tau ) = 0, \, H^{\prime}(\eta ,\tau ) = \gamma , \, I(\eta ,\tau ) = 1, \, F(\eta ,\tau ) = 1 \, at \, \eta = 0 \hfill \\ J^{\prime}(\eta ,\tau ) = 0 \, , \, H^{\prime}(\eta ,\tau ) = 0 \, , \, I(\eta ,\tau ) = 0, \, F(\eta ,\tau ) = 0 \, as \, \eta = \infty . \hfill \\ \end{gathered}$$

## Computational strategy and validation

Nevertheless, numerous semi-analytical and numerical techniques are utilizing to explore nonlinearity within the flow problems. Yet, it is significant to note here that the bvp4c method stands out as a more efficient approach. The combination of the shooting technique with this method proves to be a robust approach for ordinary differential equations solutions. The concise bvp4c method effectively handles boundary value problems with precision and efficiency. bvp4c strategy has been widely applied in commercial software such as MATLAB programing platform^19^. The computational strategy utilized in the shooting method entails transforming the given boundary value problem (BVP) stated in Eqs. (13)–(17) into an initial value problem (IVP). This modified IVP can subsequently be cracked out utilizing the bvp4c technique. Figure 1b presents the flowchart detailing the proposed methodology. Furthermore, we taken a step size of $\Delta \eta = 0.001$ to calculate the numerical solution with $\eta_{\max } = 8$, and the convergence criteria is set up to sixth decimal place. The findings demonstrate that selecting $\eta_{\max } = 8$ satisfies the effect of the boundary layer appropriately. In this approach, variables are assigned based on the order of each equation to convert nonlinear ODEs into first-order linear ODEs. The relevant variables for this purpose are as monitors:

51 $$u_{0} = y_{1} ,\;u_{0}^{\prime } = y_{2} ,\;u_{0}^{\prime \prime } = y_{3} ,\;u_{1} = y_{4} ,\;u_{1}^{\prime } = y_{5} ,\;u_{1}^{\prime \prime } = y_{6} ,\;g_{0} = y_{7} ,\;g_{0}^{\prime } = y_{8} ,\;h_{0} = y_{9} ,\;h_{0}^{\prime } = y_{10}$$

The aforementioned variables are incorporated into Eqs. (13)–(17). The bvp4c method is applied to simplify the graded equations as outlined below:

52 $$y_{3}^{\prime } = \frac{{y_{2}^{2} - \left( {y_{1} + y_{4} } \right)y_{3} - \lambda \left( {y_{7} + \alpha y_{9} } \right)}}{{\left( {1 - \frac{1}{6}\beta_{1} y_{3} } \right)^{n - 1} \left( {1 - \frac{1}{6}\beta_{1} \left( {1 + n} \right)y_{3} } \right)}}$$

53 $$y_{6}^{\prime } = \frac{{y_{5}^{2} - \left( {y_{1} + y_{4} } \right)y_{6} }}{{\left( {1 - \frac{1}{6}\beta_{2} y_{6} } \right)^{n - 1} \left( {1 - \frac{1}{6}\beta_{2} \left( {1 + n} \right)y_{6} } \right)}}$$

54 $$y_{8}^{\prime } = - \Pr \left( {\left( {y_{1} + y_{4} } \right)y_{8} + Ec\left( {1 - \frac{1}{6}\beta_{1} y_{3} } \right)^{n} y_{3}^{2} + Nb\left( {y_{8} y_{10} + \frac{Nt}{{Nb}}y_{8}^{2} } \right)} \right)$$

55 $$y_{10}^{\prime } = Sc\left( {\sigma y_{9} \left( {1 + \delta y_{7} } \right)^{m} \exp \left( {\frac{ - E}{{1 + \delta y_{7} }}} \right) - \left( {y_{1} + y_{4} } \right)y_{10} } \right) - \left( {\frac{Nt}{{Nb}}} \right)y_{8}^{\prime } .$$

In line with the following initial conditions:

56 $$\begin{aligned} & y_{1} \left( 0 \right) = 0,\;y_{2} \left( 0 \right) = 1,\;y_{4} \left( 0 \right) = 0,\;y_{5} \left( 0 \right) = \gamma ,\;y_{7} \left( 0 \right) = 1,\;y_{9} \left( 0 \right) = 1 \\ & y_{2} \left( \infty \right) = 0,\;y_{5} \left( \infty \right) = 0,\;y_{7} \left( \infty \right) = 0,\;y_{9} \left( \infty \right) = 0. \\ \end{aligned}$$

Furthermore, to enhance the study and facilitate learning of the bvp4c numerical method, a flowchart is depicted via Fig. 1b.

**Figure 2:**
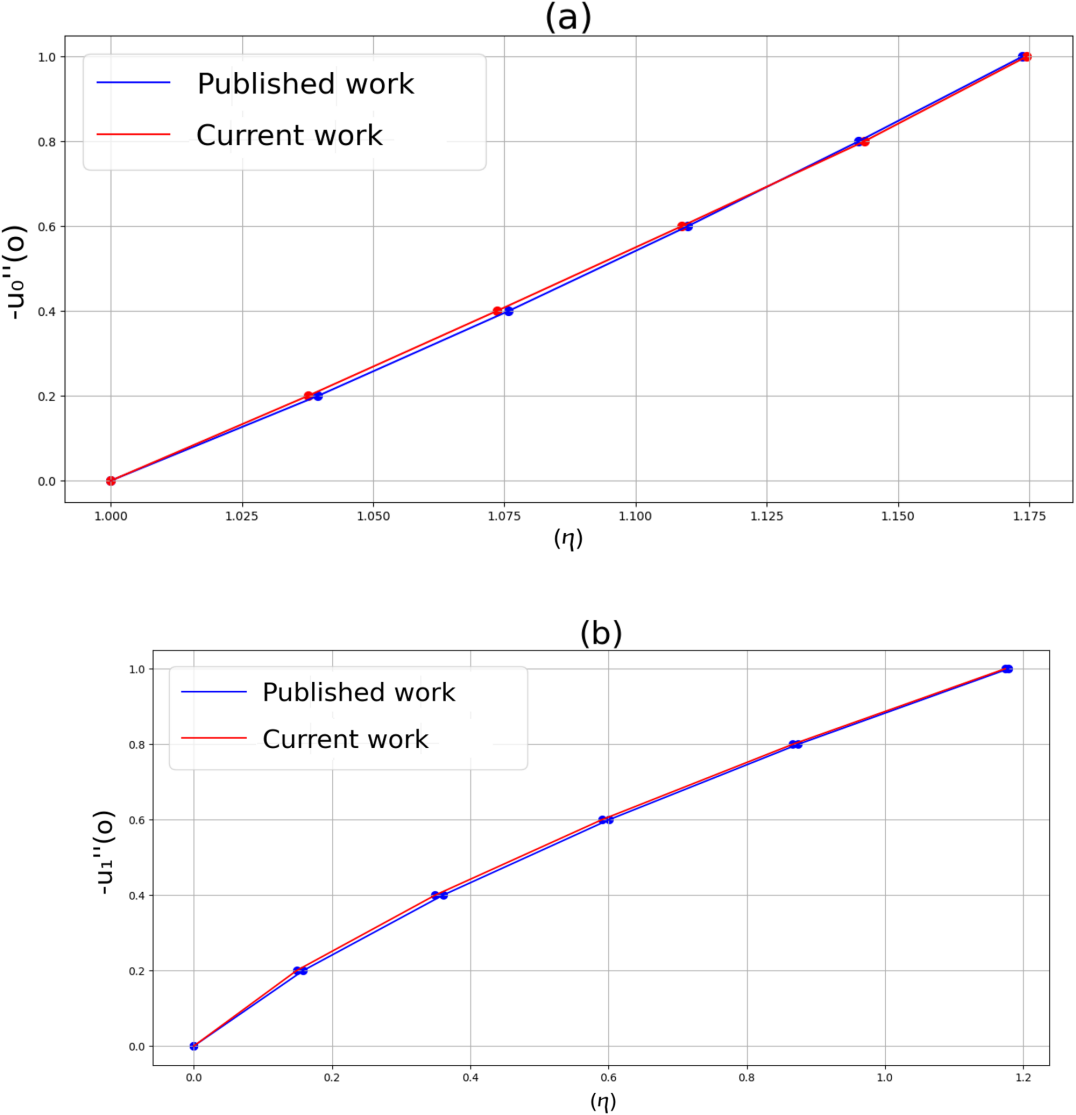
(**a**, **b**) A comparative analysis between the current findings and previously published data^48^.

After establishing the mathematical model for incompressible Sutterby nanofluid flow, which takes into consideration the impact of viscous flow features, the next step involves developing a computational algorithm to solve these governing equations. Before initiating any simulations, it is essential to verify the precision of the code. This section's objective is to delineate the authentication of the adopted method and to compare the results acquired with those already recorded in the existing literature. The accuracy and trustworthiness of the existing nonlinear computations are confirmed through a comparison of its solutions with the prior research conducted by Ariel^48^. Figure 2a,b demonstrates a remarkable alignment between the results obtained in this study and the data documented in earlier literature.

## Dual solutions and analysis

Nanofluids offer a reliable means to enhance the heat transfer rate of conventional fluids. However, simply dispersing nanoparticles within a fluid is not enough to achieve this. Limitations exist within the chemical and thermophysical characteristics of the fluid, and nanofluids are employed to bridge this gap. Furthermore, a specialized thermophysical relationship between viscosity and thermal conductivity is employed in this study to significantly improve the conduction performance of the fluids. The model problem under consideration involves a stretching/shrinking surface, where turbulence reduction is critical for stability. To address this, a magnetic field is introduced into the flow field. The flow pattern is considered to be steady. The solution to the proposed problem is computed using bvp4c techniques, and its validity has been established through a review of existing literature. Notably, this section focuses on the primary concern of the flow field and thermal analysis, with the model parameters playing a pivotal role in achieving this goal.

**Figure 3:**
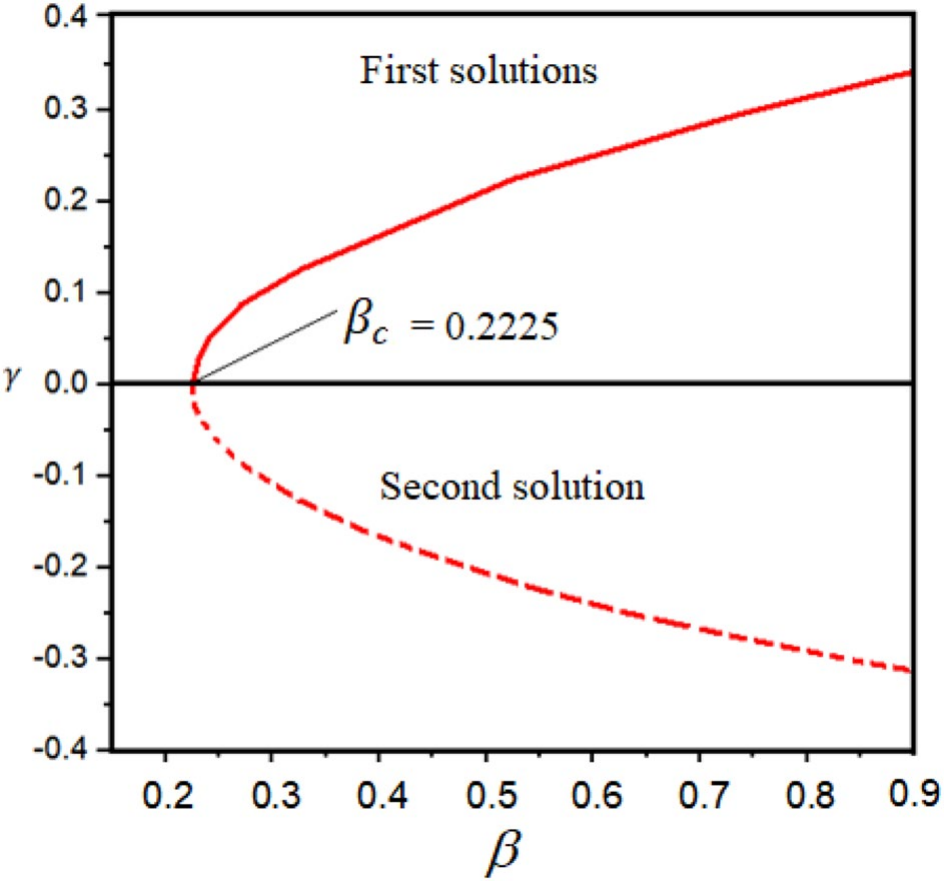
Dual solutions presentation regarding $\beta$.

Due to stretching and shrinking sheet, dual solutions occur for some physical parameters. Therefore, the stability analysis is implemented as discussed in “Stability analysis” section. The linearized Eqs. (46)–(50) are numerically solved using the bvp4c approach. The graphical illustration of the multiple solutions is provided in Fig. 3. The smallest positive eigenvalues demonstrate a stable, while the negative eigenvalues report unreliable solutions for the suction parameter, as reported by Merkin^46^ and Wiedman et al.^47^. The common point that combines the first and second branches is known as the critical point i.e., $\beta_{c} = 0.2225$ as shown in Fig. 3. The stability analysis is significantly implemented to analyses the stable branch when multiple branches occur.

**Figure 4:**
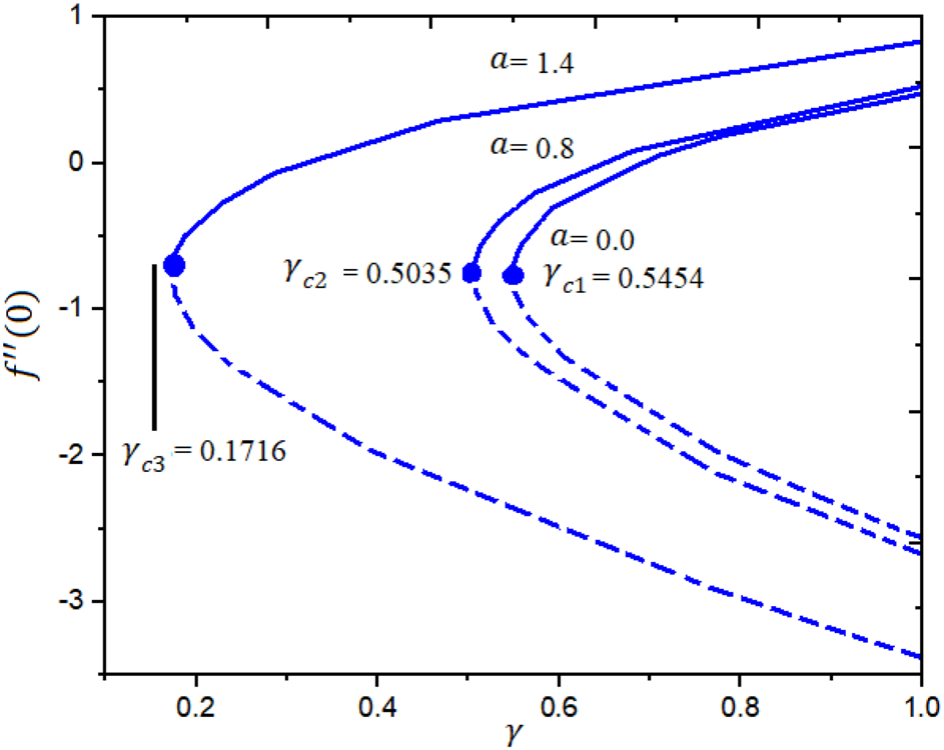
Inspiration of *a* on $f^{\prime \prime } (0)$.

**Figure 5:**
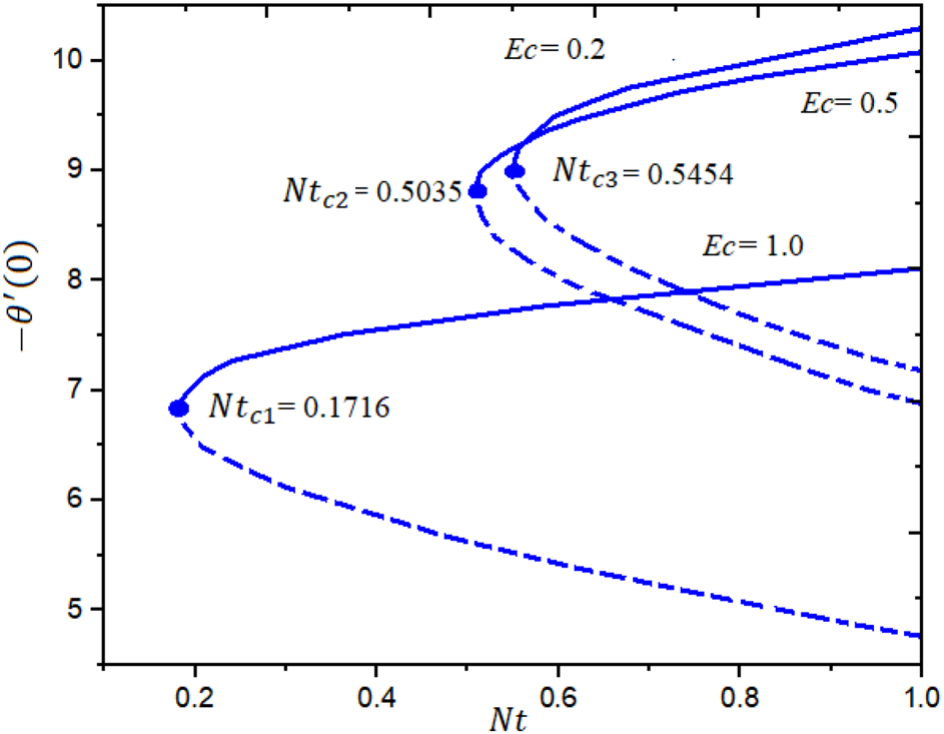
Inspiration of *Ec* on $- \theta^{\prime } (0)$.

**Figure 6:**
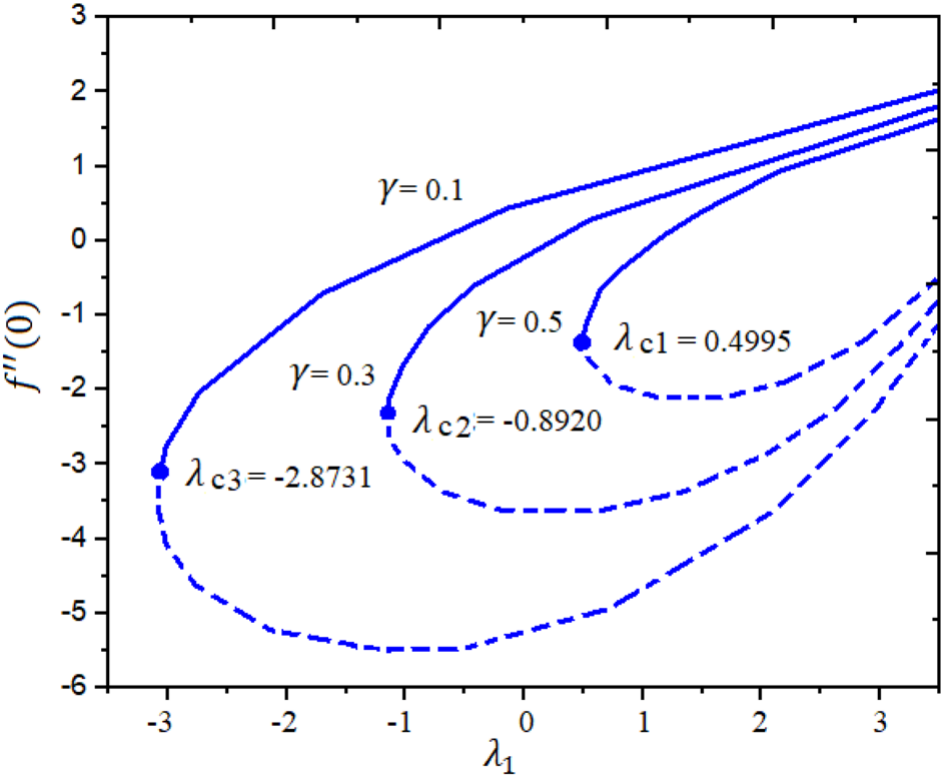
Inspiration of $\gamma$ on $f^{\prime \prime } (0)$.

**Figure 7:**
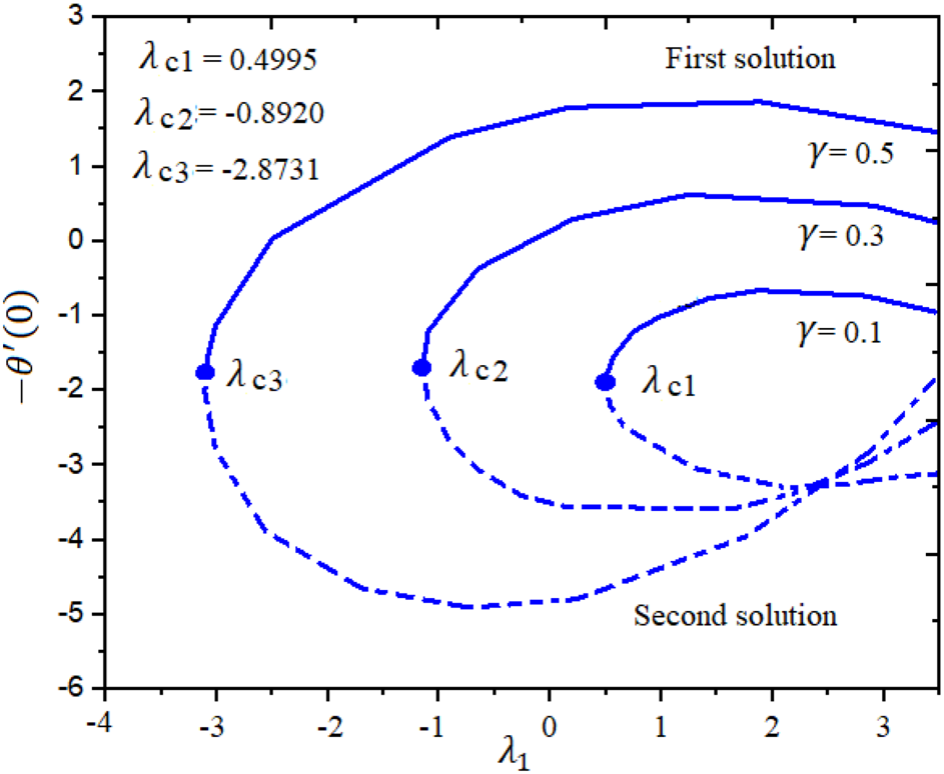
Inspiration of $\gamma$ on $- \theta^{\prime } (0)$.

**Figure 8:**
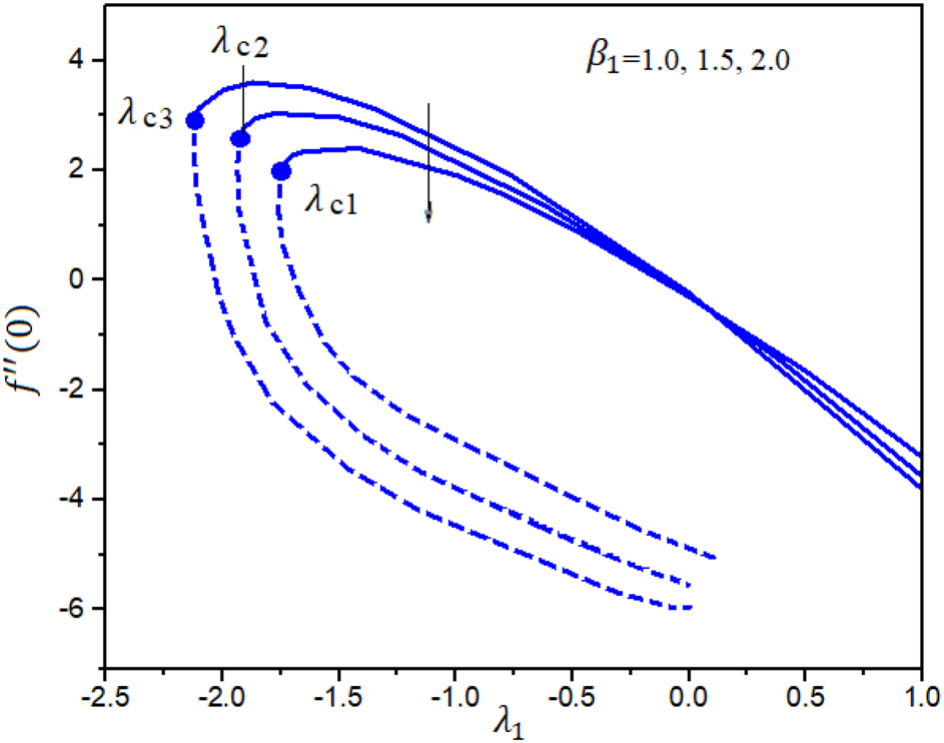
Inspiration of $\beta_{1}$ on $f^{\prime \prime } (0)$ .

**Figure 9:**
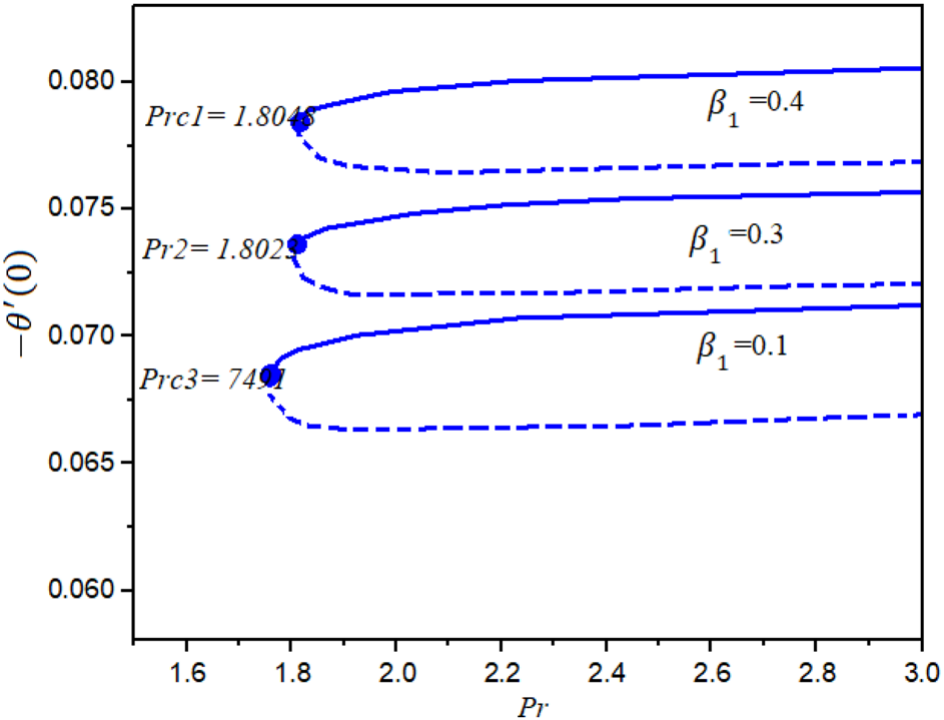
Inspiration of $\beta_{1}$ on $- \theta^{\prime } (0)$.

Figures 4 and 5 represent the impact of $a$ and $Ec$ on the Local Skin Friction (LSF) and Local Nusselt Number (LNN) regarding $\gamma$ and $Nt$, respectively. Dual exploration has been explored for $a$ and. The solid line reports the first branch while the dashed line identifies the second solutions. The critical quantities for $a = 0.0, 0.8,$ and $1.4$ are $\gamma_{c1} = 0.5454$, $\gamma_{c2} = 0.5035$, and $\gamma_{c3} = 0.1716$, respectively. It is interesting to note here that the LSF enhances for the growing values of $a$ in the first solution while declining in the second solution, as publicized in Fig. 4. Furthermore, the heat rate drops in the first as well as second solutions as the quantities of $Ec$ are increased as depicted in Fig. 5. The corresponding critical values for $Ec$ = 0.2, 0.5 and 1.0 are $Ec_{c1} = 0.5454$, $Ec_{c2} = 0.5035$, and $Ec_{c3} = 0.1716$, respectively. The relations between $\gamma$ and stretching/shrinking sheet factor $\lambda_{1}$ on the LSF and LNN is reported in Figs. 6 and 7, correspondingly. It is investigated that the LSF enhances with the growing magnitudes of $\gamma$ and $\lambda_{1}$ while reverse effect is observed for LNN. In Fig. 6 it is significant to note that dual expression occurs in the second branch. In the range $- 3 \le \lambda_{1} < 2$, decline behavior is observed for the LNN while in the range $2 \le \lambda_{1} \le 3$ increasing behavior is reported. Additionally, the impact of $\beta_{1}$ on the LSF and LNN is displayed in Figs. 8 and 9, respectively ragrading stretching/shrinking parameter $\lambda_{1}$ and Prandtl number $Pr$. It is interesting to note here that as the $\beta_{1}$ is increased, the profiles of $f^{\prime \prime } \left( 0 \right)$ decelerated while the profile $- \theta^{\prime } \left( 0 \right)$ is increased in the first branch, which gives admirable settlement with the outcomes described by Waini et al.^49^. This analysis shows a 3.8% increase in heat rate as $\beta_{1}$ enhanced. This analysis proved that when $\beta_{1}$ is enhanced, the thermal efficiency of the host fluid enhances. Also, it is reported that as the wedge surface moves at $\lambda_{1} = 1.0$, the LSF become zero i.e., $f^{\prime \prime } \left( 0 \right) = 0,$ due to no FDF on the wedge heating sheet convectively.

**Figure 10:**
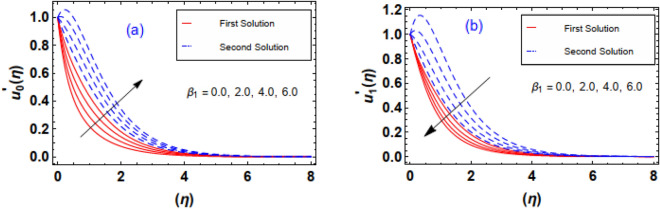
(**a**, **b**) Outlines of $\beta_{1}$ via velocities profiles.

**Figure 11:**
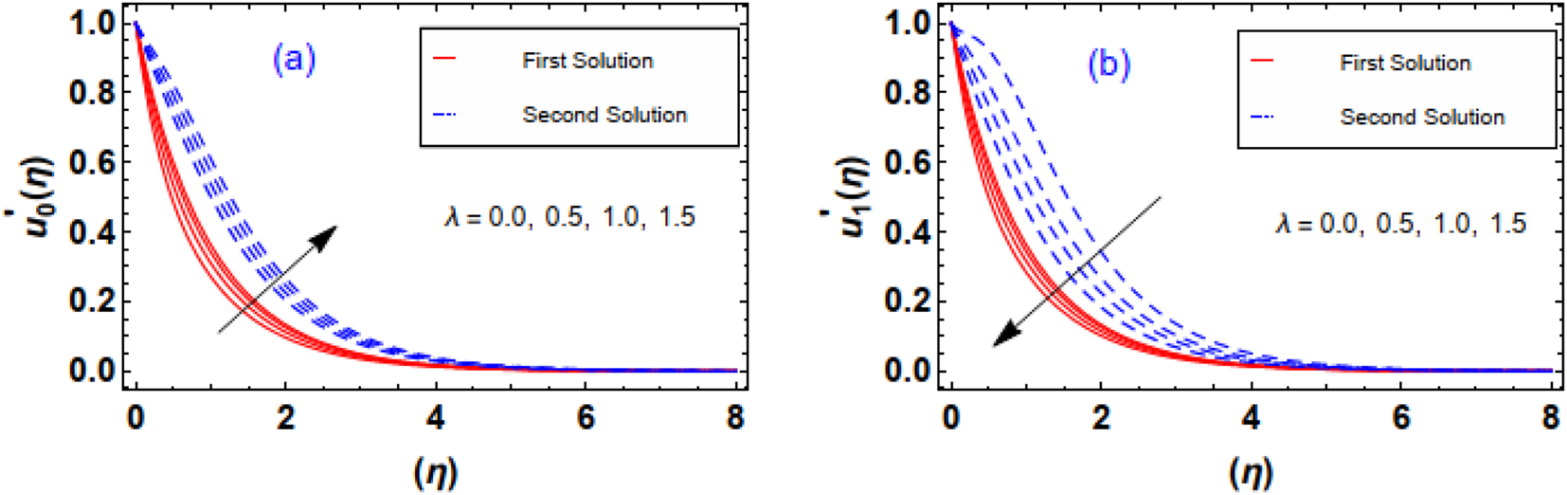
(**a**, **b**) Outlines of $\lambda$ via velocities profiles.

**Figure 12:**
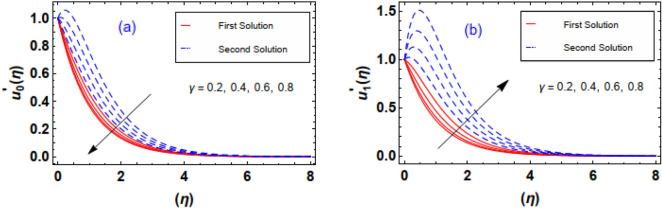
(**a**, **b**) Outlines of $\gamma$ via velocities profiles.

Figure 10a,b exhibits the flow nature of the velocities $u_{0}^{\prime } \left( \eta \right)\;and\;u_{1}^{\prime } \left( \eta \right)$ profiles for the Sutterby fluid parameter $\beta_{1}$. It is discerned that the both fluid profiles display opposite behavior when the $\beta_{1}$ is raised. Actually, the elevating data of $\beta_{1}$ diminishes the internal resistance of the fluid layers and resultantly, fluid velocity distribution $u_{0}^{\prime } \left( \eta \right)$ grows and $u_{1}^{\prime } \left( \eta \right)$ profile drops. The contribution convection fluid factor $\lambda$ via $u_{0}^{\prime } \left( \eta \right)\;and\;u_{1}^{\prime } \left( \eta \right)$ is estimated through Fig. 11a,b. Clearly, it is measured that greater exalting estimation of convection parameter $\lambda = 0.0, \, 0.5, \, 1.0, \, 1.5$ enhances the fluid velocity $u_{0}^{\prime } \left( \eta \right)$ however an inverse trend in the fluid velocity $u_{1}^{\prime } \left( \eta \right)$ profile is witnessed. Physically, such scenario is noticed for the increasing positive data of $\lambda$ correspond to assisting flow which improve velocity field $u_{0}^{\prime } \left( \eta \right)$. It is important to pointed out that the fluid convection factor $\lambda$ classifies as forced convection flow for $\lambda = 0$ and free convection observed in case of larger estimation of convection parameter i.e. $\lambda = \infty$. Furthermore, in case of the convection factor $\lambda$ is greater than zero, it indicates flow assisting flow, whereas when $\lambda$ is less than zero, it implies flow opposing flow. Aftermath of incrementing data of ratio of stretching rates parameter $\gamma = 0.2, \, 0.4, \, 0.6,{ 0}.8$ is interpreted through Fig. 12a,b. The behavior velocity $u_{1}^{\prime } \left( \eta \right)$ is augmented due to increasing values of $\gamma$ parameter while an opposite tendency in flow field of nanofluid $u_{0}^{\prime } \left( \eta \right)$ is anticipated in these outlines. In reality, higher estimation of $\gamma$ attribute in the acceleration of extension rate in the y − direction. Consequently, the flow rate profile $u_{1}^{\prime } \left( \eta \right)$ augmented in y − direction.

**Figure 13:**
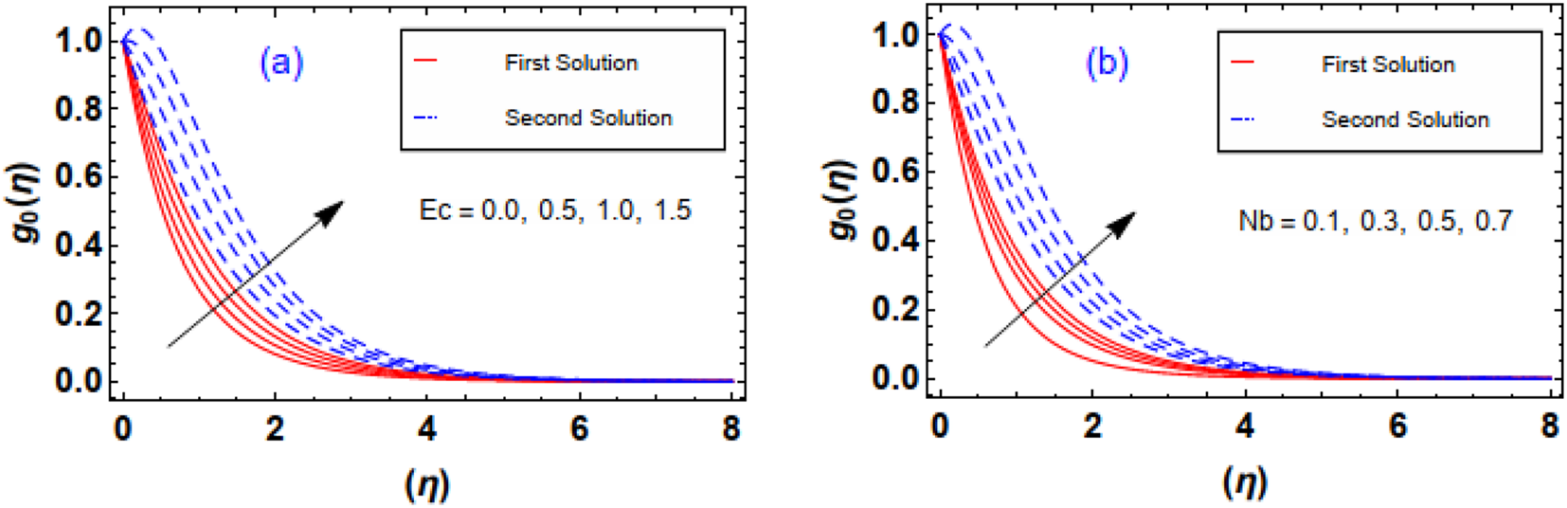
(**a**, **b**) Impact of $Ec\;and\;Nb$ via temperature gradient.

**Figure 14:**
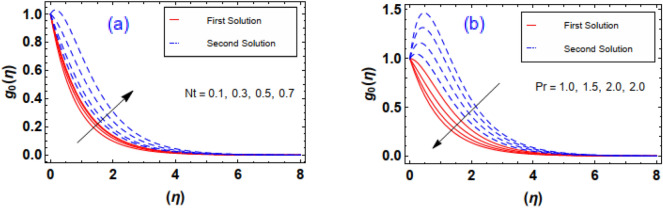
(**a**, **b**) Impact of $Nt\;and\;\Pr$ via temperature gradient.

The outcomes of $Ec \, and \, Nb$ parameters via the thermal field $g_{0} \left( \eta \right)$ is sketched through Fig. 13a,b. It is apparent that the profile and respective thermal layer are incremented with greater amplification of the parameter. Physically, magnifying enhances the internal kinetic energy fluid nanoparticles. Thus, the fluid temperatureswell up. Figure 13b illuminates the aftermath of the particles diffusion factor on heat gradient. As anticipated, increases. Physically, increment inparameter accelerate the diffusion rate inside the fluid particles, and at this stage, particles acceleration and its impact against the fluid play a significant role regarding the heat transmission mechanism. As expected, raise inincreases the disordered movement of fluid particles. Therefore, the kinetic energy of the entire fluid uplifts and ultimatelyincreases. Figure 14a,b explains variations in subject to. As estimated, temperature profile uplifts through higher estimation of thermophoresis. Physically, thermophoresis force swells when thermophoresis is augmented. In such scenario the exalting behavior of thermophoretic force helps to enhance the disorder movement of liquid particles by hotter region towards colder region. Thus, increase. Impact of on thermal field curves is exposed in Fig. 14b. As witnessed, a reduction behavior in noticed against the expending estimation of the number. According to the physical insight of, the increasing Pr is a reciprocal impact to the thermal diffusivity of the liquid. Therefore, expanding nature of number uplift the diffusivity. Thus, both temperature and thermal boundary layer distribution reduces.

**Figure 15:**
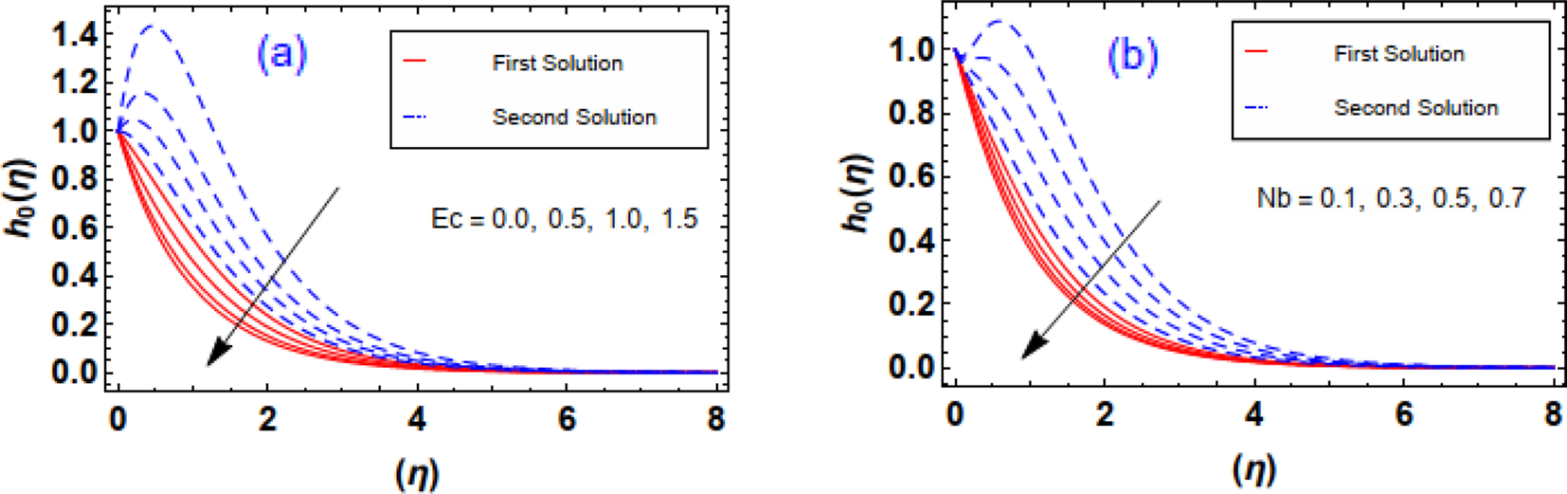
(**a**, **b**) Effect of $Ec\;and\;Nb$ against concentration profiles.

**Figure 16:**
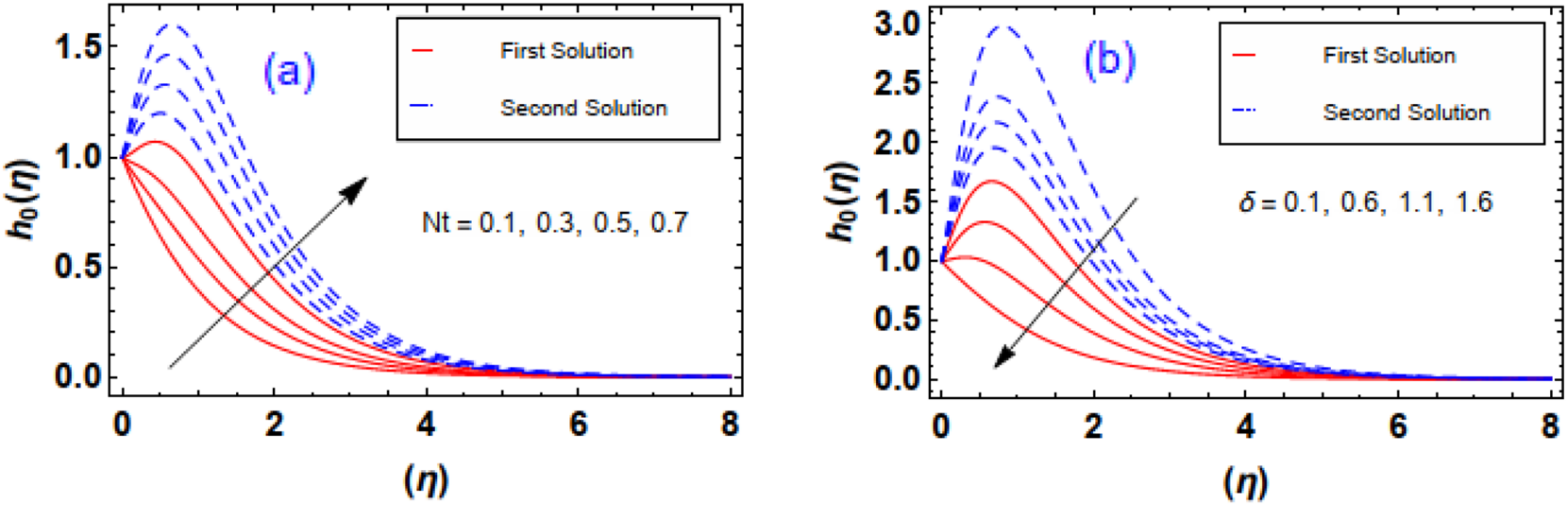
(**a**, **b**) Effect of $Nt\;and\;\delta$ against concentration profiles.

**Figure 17:**
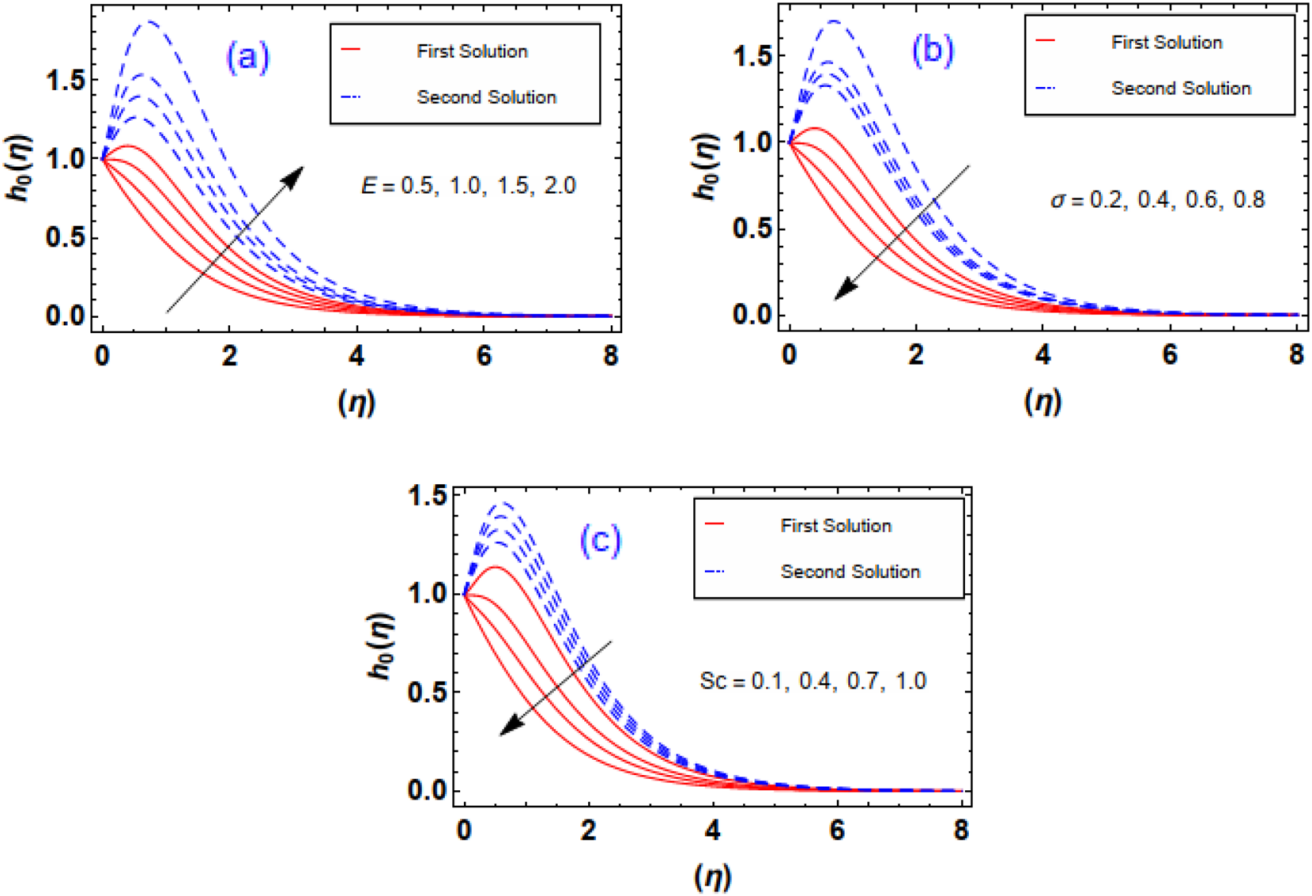
(**a**–**c**) Effect of $E,\sigma \;and\;Sc$ against concentration profiles.

**Figure 18:**
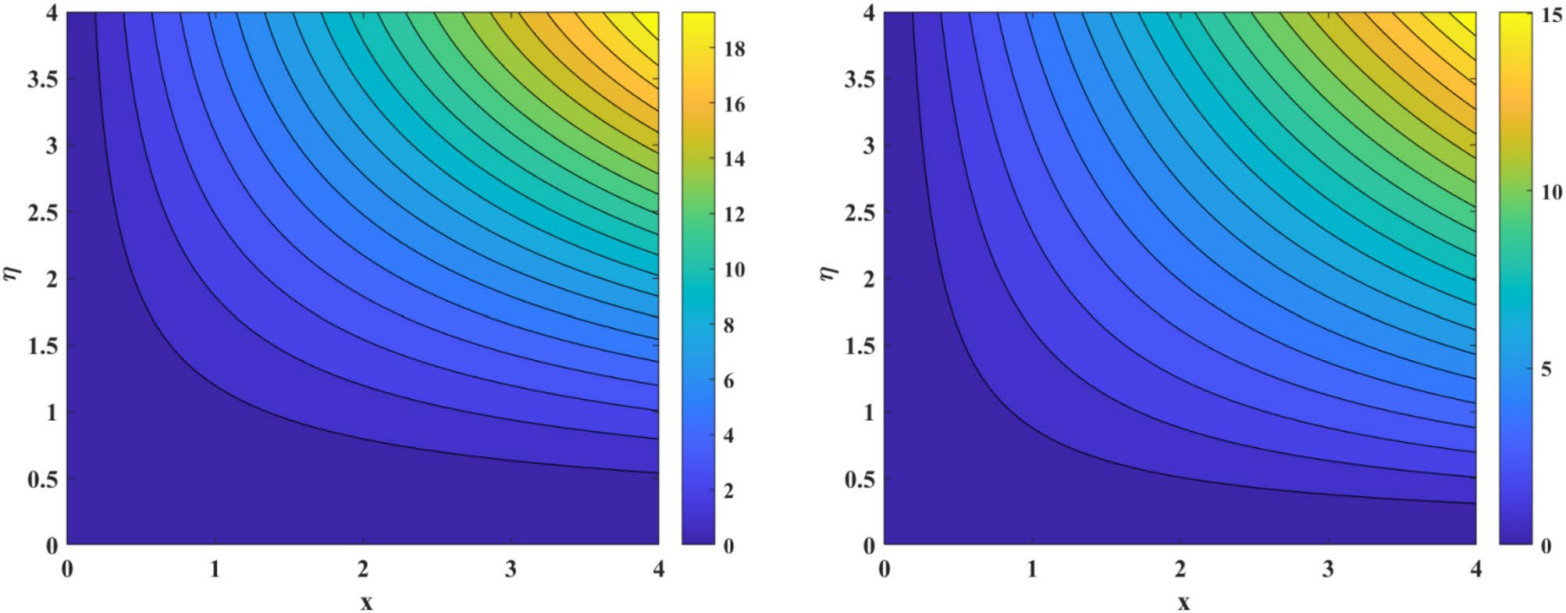
Streamlines for $\beta_{1} = 2$ and $\beta_{1} = 4$.

**Figure 19:**
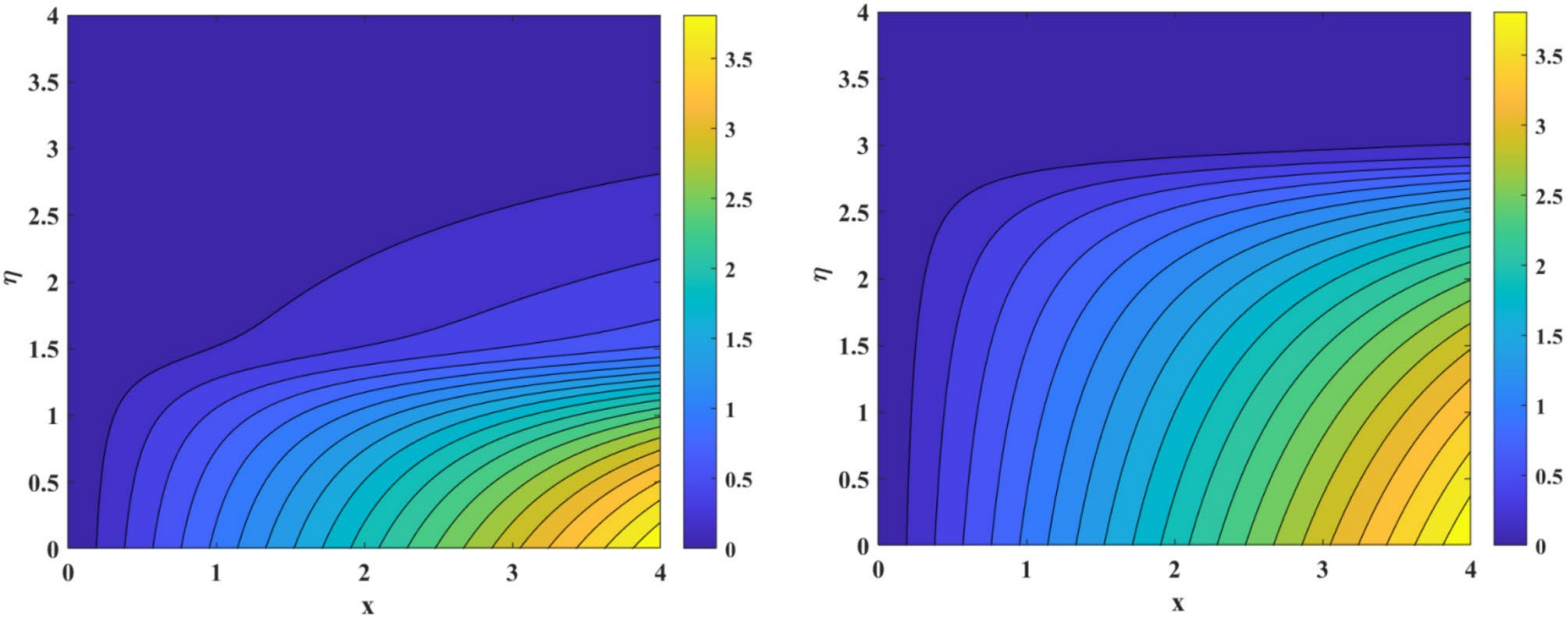
Isothermal flow for *Nt* = 0.2 and *Nt* = 1.2.

The aftermath of Eckert number and Brownian diffusion on concentration is probed through Fig. 15a,b. It is reported that the concentration of nanoparticle is marked down for the higher rating of parameter highlighted through Fig. 15a. Figure 15b interfaces the impacton fluid distribution. Clearly, it is mentioned that escalating data of aggrandizes the distribution nanoparticles concentration. Physically, expanding nature of uplifts the diffusion process and disordered movement by which nanoparticles move with diverse direction with random particles movements due to the Brownian effect. Subsequently, concentration profile and corresponding thickness of the fluid layer falls. The characteristics of and on are observed through Fig. 16a,b. It is evaluated that concentration is directly influenced by the higher estimation of the thermophoresis force parameter. Physically, uplifting data of increases the count of nanoparticle due to which molecules move from hotter to cooler surfaces. As a result, increases outlined via Fig. 16a, whereas differing behaviors via greater estimations of values is witnessed in Fig. 16b. Figure 17a–c explains the variations in regarding to swelling values of activation energy, Schmidt number, and chemical reaction factors. As estimated, the fluid concentration profile is enlarged due to the expanding supplement of energy parameter shown through Fig. 17a. The impressions of are sketched in Fig. 17b. Here, it is looked over that the greater approximation of σ replicates in the reduction of distribution. The reason for such scenario is that expending values of implies larger destruction due to the chemical process which dissolves liquefies liquid species efficiently. Thus, reduces. Figure 17c explains Schmidt number effect against . Actually, Schmidt number and mass diffusivity are inversely related. Hence, higherowns a reduction in concentration outline. Physically, mass diffusion declines when Schmidt number uplifts. Consequently, decreases. Streamlines are plotted for two different values of $\beta_{1} = 2$ and $\beta_{1} = 4$ as shown in Fig. 18. For different values of $M$ streamlines are declined. The isothermal flow for the given model is explored in Fig. 19 using different values of radiation factor. It is revealed that as the value of $R_{d}$ increases from $Nt$ = $0.2$ to $Nt$ = $1.2$, the isothermal flow enhances.

## Closing remarks

The novel aspect of present analysis is that, it scrutinizes the incompressible steady flow of 3D Sutterby nanofluid across a bidirectional extended surface, including the influences of activation energy, mixed convection, viscous dissipation, and chemical reaction. Effects of thermophoresis diffusion and Brownian movement motion is deliberated in the model problem. The modeled resulting nonlinear PDEs are diminished into a dimensionless system of ODEs using resemblance replacements. Subsequently, the obtained set of differential equations is resolved statistically^49–53^. The findings of this study yield several conclusions as follows:

- Dual exploration has been explored for $a$ and . The critical quantities for $a = 0.0, 0.8,$ and $1.4$ are $\gamma_{c1} = 0.5454$, $\gamma_{c2} = 0.5035$, and $\gamma_{c3} = 0.1716$, respectively.
- The corresponding critical values for $Ec$ = 0.2, 0.5 and 1.0 are $Ec_{c1} = 0.5454$, $Ec_{c2} = 0.5035$, and $Ec_{c3} = 0.1716$, respectively.
- It is interesting to note here that the LSF enhances for the growing quantities of $a$ in the first solution while declining in the second solution.
- The heat rate reductions in the first and second solutions is reported as the quantities of $Ec$ are increased.
- As notice the fluid velocity along x-direction increases for greater estimation of Sutterby fluid and mixed convection parameters while opposite trend noticed for y-direction flow stream.
- The energy profile increased by uplifting the magnitude of Eckert number, Brownian, and thermophoresis parameters but decreases by enhancing the values of Prandtl number.
- The concentration outline decremented with expanding nature of Brownian parameter, and Eckert number, while increases by increasing thermophoresis diffusion parameter and Arrhenius energy parameter.

This study can be improved by adding the slip effect for hybrid nanofluid with stability analysis.

## Data Availability

The datasets used and/or analysed during the current study available from the corresponding author on reasonable request.
